# High ammonia adsorption in copper-carboxylate materials: host–guest interactions and crystalline–amorphous–crystalline phase transitions†

**DOI:** 10.1039/d4cc02604g

**Published:** 2024-09-16

**Authors:** Wanpeng Lu, Yinlin Chen, Zi Wang, Lixia Guo, Jin Chen, Yujie Ma, Weiyao Li, Jiangnan Li, Meng He, Mengtian Fan, Alena M. Sheveleva, Floriana Tuna, Eric J. L. McInnes, Mark D. Frogley, Philip A. Chater, Catherine Dejoie, Martin Schröder, Sihai Yang

**Affiliations:** a Department of Chemistry, University of Manchester Manchester M13 9PL UK M.Schroder@manchester.ac.uk; b College of Chemistry and Molecular Engineering, Beijing National Laboratory for Molecular Sciences, Peking University Beijing 100871 China Sihai.Yang@pku.edu.cn; c Photon Science Institute, University of Manchester Manchester M13 9PL UK; d Diamond Light Source, Harwell Science Campus Oxfordshire OX11 0DE UK; e European Synchrotron Radiation Facility Grenoble 38043 France

## Abstract

We report the high NH_3_ uptake in a series of copper-carboxylate materials, namely MFM-100, MFM-101, MFM-102, MFM-126, MFM-127, MFM-190(F), MFM-170, and Cu-MOP-1a. At 273 K and 1 bar, MFM-101 shows an exceptional uptake of 21.9 mmol g^−1^. The presence of Cu(ii)⋯NH_3_ interactions and changes in coordination at the [Cu_2_(O_2_CR)_4_] paddlewheel are analysed and discussed.

## Abstract 2

High NH_3_ uptake is observed in a series of paddlewheel [Cu_2_(O_2_CR)_4_] metal–organic frameworks.

## Graphical abstract

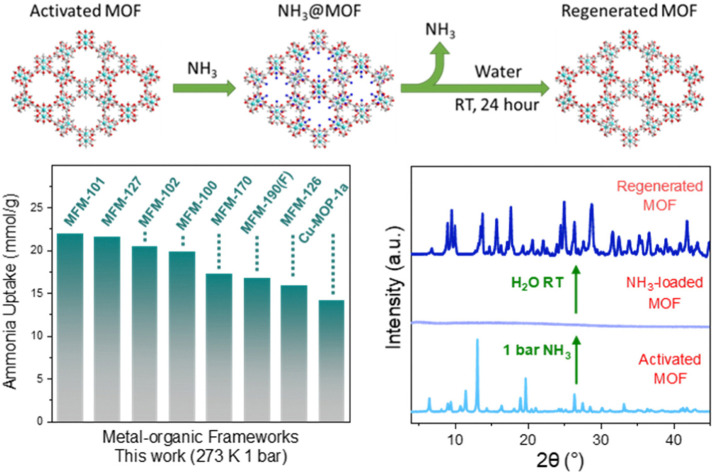

Ammonia (NH_3_) is an essential feedstock for the manufacture of fertilizers and pharmaceuticals, and for proton transport in fuel cells.^1^ NH_3_ can be compressed for transport to a liquid at ∼10 bar and room temperature or by cooling to −33 °C at atmospheric pressure. Due to its high gravimetric and volumetric hydrogen densities and potential clean combustion products (ideally N_2_ and H_2_O only), NH_3_ is also considered as a potential hydrogen storage medium and renewable liquid fuel.^2^ However, the toxicity and caustic nature of NH_3_ means that it is necessary to develop safe and efficient materials for NH_3_ storage.^3,4^ Porous materials for the physical or chemical adsorption of NH_3_ are regarded as promising hosts that are capable of high energy efficiency and low operational cost.^5^ Conventional porous materials generally show low NH_3_ adsorption capacities, for example, up to 11.4 mmol g^−1^ in Amberlyst15.^6^ Metal–organic framework (MOF) materials have attracted much interest for NH_3_ adsorption.^7^ Their superior surface area, controllable pore geometry, and flexibility to incorporate acidic and basic functional groups and open metal sites enable further enhancement of their adsorption performance.^8^ Recently, MOFs have shown high NH_3_ uptakes: 23.5 mmol g^−1^ in Ni_acryl_TMA incorporating BTDD (bis(1*H*-1,2,3,-triazolo[4,5-*b*],-[4′,5′-*i*])dibenzo-[1,4]dioxin) bridging ligands, and 19.8 mmol g^−1^ in [Cu_2_Cl_2_(BBTA)] [H_2_BBTA= 1*H*,5*H*-benzo(1,2-*d*:4,5-*d*′)bistriazole] at 298 K and 1 bar.^9^ Reversible adsorption of NH_3_ has also been observed in a series of ultra-stable MOFs, MFM-300(M) (M = Cr, Fe, V^III^, V^IV^), showing uptakes of up to 17.3 mmol g^−1^ at 273 K and 1 bar.^10^

MOFs incorporating open metal sites can demonstrate preferential binding of gases, but they often exhibit poor stability upon adsorption of reactive gases, such as NH_3_, leading to irreversible desorption and/or structural collapse.^11^ NH_3_-induced structural transformation has been observed recently in [Cu(CYHDC)] (CYHDC^2−^ = *trans*-1,4-cyclohexanedicarboxylate) with a reported crystalline phase transition from 3D porous framework to 1D coordination polymer.^12^ Here, we report the high adsorption of NH_3_ in a series of Cu(ii)-based MOFs and metal–organic polyhedra (MOP) and the comprehensive investigation of the mechanisms of adsorption by *in situ* synchrotron X-ray powder diffraction (SPXRD), single crystal synchrotron Fourier transform infrared (FTIR) micro-spectroscopy, X-ray pair distribution function (XPDF) and electron paramagnetic resonance (EPR) spectroscopy.

MFM-100, MFM-101, MFM-102, MFM-126, MFM-127, MFM-190(F), MFM-170, and Cu-MOP-1a were synthesised based upon our previously reported methods (Fig. S1, ESI†).^13^ All of these materials are based upon [Cu_2_(O_2_CR)_4_] paddlewheel nodes. The isotopological MFM-100, MFM-101 and MFM-102 are composed of di-, tri-, and tetra-phenyl tetracarboxylate linkers, respectively, and exhibit interconnected cages with pore size dimensions of 12 × 12 × 12 Å, 12 × 12 × 22 Å and 12 × 12 × 28 Å, respectively. Another group of isostructural MOFs, MFM-126 and MFM-127, feature pyrimidine-dicarboxylate ligands and each afford two different types of cages (12 × 12 × 12 Å and 10 × 10 × 15 Å; 12 × 12 × 13 Å and 12 × 12 × 16 Å, respectively). MFM-190(F) incorporates a fluoro-functionalised pyridyl ring and shows two cages with dimensions of 18 × 18 × 14 Å and 18 × 18 × 24 Å. MFM-170 is constructed with a pyridyl-tetracarboxylate linker and has three interconnected cages (12 × 12 × 12 Å, 12 × 12 × 16 Å and 16 × 16 × 22 Å). Cu-MOP-1a incorporates a tripodal tricarboxylate ligand and has three different types of spherical cages with diameters of 5, 6 and 15 Å. The gravimetric NH_3_ adsorption isotherms for these materials were recorded at 273–298 K up to 1 bar, and the corresponding volumetric uptakes measured (Table S1, ESI†). At 273 K and 1 bar, the isotherms show NH_3_ uptakes ranging from 14.2 to 21.9 mmol g^−1^ (Fig. 1a and b). Broad hysteresis loops and high residues of NH_3_ (up to 14.6 mmol g^−1^ for MFM-127) are observed for all materials upon pressure-swing desorption (Fig. S2 and S3, ESI†), indicating the presence of strong host–guest interactions in these systems. Heating up to 150 °C under vacuum leads to removal of all NH_3_. For all the studied MOFs, loss of crystallinity is observed upon adsorption of NH_3_ at 1 bar characterized by loss of peaks by PXRD (Fig. S4, ESI†). However, MFM-100, MFM-101 and MFM-102 show a degree of structural stability towards adsorption of NH_3_ up to 50 mbar (Fig. S5, ESI†) and were of special interest due to their high uptakes of NH_3_ at 1 bar, 19.8, 21.9 and 20.4 mmol g^−1^ at 273 K and 15.7, 14.9 and 13.7 mmol g^−1^ at 298 K. It should be noted that MFM-100 demonstrates much the best structural stability up to 50 mbar in comparison to MFM-101 and MFM-102, probably due to greater rigidity of the framework incorporating a shorter linker.

**Fig. 1:**
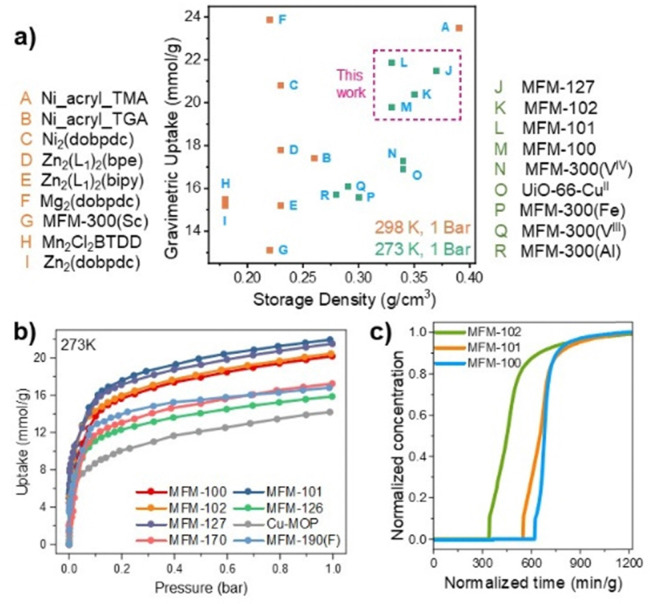
(a) Plot of gravimetric uptake and storage densities for NH_3_ in selected MOFs at 1 bar: orange 298 K, green 273 K (Tables S4–S6, ESI†). (b) Adsorption isotherms for NH_3_ in MFM-100, MFM-101, MFM-102, MFM-126, MFM-127, MFM-190(F), MFM-170 and Cu-MOP-1a at 273 K. (c) Breakthrough curves for NH_3_ (1000 ppm diluted in He) through fixed beds of MFM-100, MFM-101 and MFM-102 at 298 K at 1.0 bar.

Despite the loss of crystallinity upon exposure to NH_3_ at 1 bar, MFM-100, MFM-101 and MFM-102 maintained their NH_3_ uptake over five cycles of pressure-swing adsorption–desorption up to 0.15 bar NH_3_ at 298 K (Fig. S6, ESI†). The loss of crystallinity up to 1 bar NH_3_ (Fig. S4, ESI†) suggests loss of long-range structural periodicity in these materials rather than full framework collapse (see below). Relatively strong host–guest interactions are suggested by the measured high heats of adsorption (30 to 90 kJ mol^−1^, Fig. S7, ESI†). Temperature-programmed desorption of NH_3_ (NH_3_-TPD) showed a main desorption peak for MFM-100 at 100 °C, while for MFM-101 and MFM-102 this peak is broadened and shifted towards higher temperatures (100–250 °C) (Fig. S8, ESI†). To study further the performance of these materials for capture of NH_3_ at low concentrations (1000 ppm), conditions under which these materials are stable and remain crystalline, breakthrough experiments were performed at 298 K. These show dynamic uptakes of 3.3, 4.1 and 4.5 mmol g^−1^, respectively, for MFM-100, MFM-101 and MFM-102 (Fig. 1c), consistent with the observed isothermal uptakes at 1 mbar (3.6, 4.4 and 4.9 mmol g^−1^, respectively, Table S5, ESI†). The highest dynamic uptake was observed for MFM-102, the material with the largest pore size which allows rapid adsorption equilibrium under flow conditions.

The preferred binding domains of NH_3_ in MFM-100 were determined by the Rietveld refinement of *in situ* SPXRD data of gas-loaded MFM-100 (Fig. S9 and S10, ESI†). At 50 mbar of NH_3_, conditions under which the material is stable, three binding sites are observed in MFM-100·4.4NH_3_ [Cu_2_(C_16_O_8_H_6_)·4.4NH_3_] (Fig. 2a–c), corresponding to a crystallographic uptake of 9.69 mmol g^−1^. This is consistent with the isothermal uptake of 10.8 mmol g^−1^ at 50 mbar. All three binding sites are found within the rhombic cage, where the predominant position, Site I [occupancy= 0.80(3)] is bound to Cu(ii) [Cu⋯N̲H_3_ = 2.78(29) Å], consistent with the notable shift of ligand-field (d–d) transitions from 700 to 620 nm in the UV-vis spectrum typically observed for the formation of Cu–NH_3_ complexes (Fig. 2d).^14^ Site II [occupancy= 0.52(3)] is located towards the centre of the cage and binds to the ligand *via* hydrogen bonding [C–H_aromatic_⋯N̲H_3_ = 2.06(10) Å]. The *in situ* synchrotron FTIR spectra show that the aromatic C–H stretching modes (3055 and 3048 cm^−1^) and bending mode (840 cm^−1^) decrease in intensity with increasing NH_3_ partial pressure (Fig. 2e and Fig. S11–S13, ESI†).^15^ Only guest–guest interaction is observed for NH_3_ at site III [occupancy= 0.19(3)], with adjacent molecules in the pore exhibiting intermolecular hydrogen bonding [N^1^⋯N^2^ = 2.57(31) Å, N^2^⋯N^3^ = 2.58(48) Å] to afford efficient packing of adsorbed NH_3_ molecules in the pore.

**Fig. 2:**
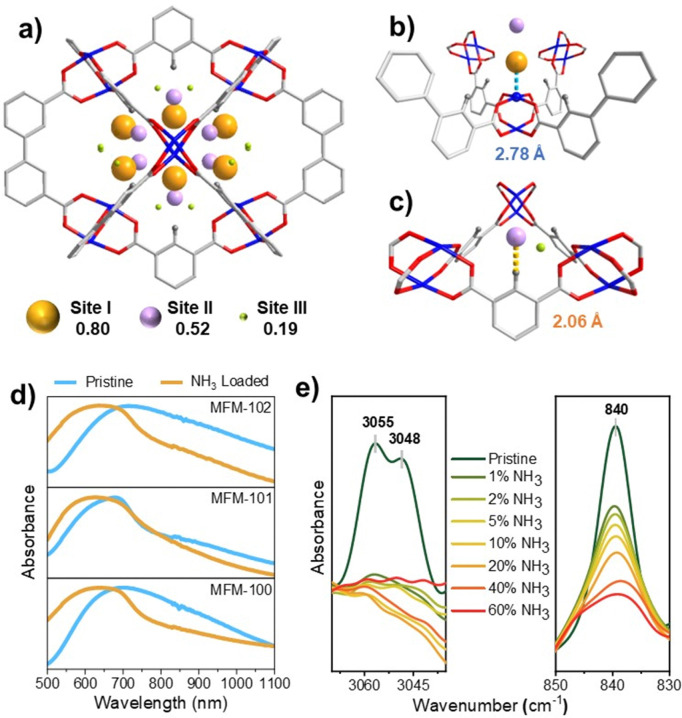
(a) Location of NH_3_ in MFM-100·4.4NH_3_ determined by SPXRD with the radius of the sphere proportional to occupancy. (b) Binding site I shows interaction of NH_3_ with open Cu(ii) site. (c) Binding site II shows aromatic C–H⋯NH_3_ hydrogen bonding. (d) UV-vis spectra of MFM-100, MFM-101 and MFM-102 upon adsorption of NH_3_. (e) *In situ* synchrotron FTIR spectra for single crystals of MFM-101 at various partial pressures of NH_3_ (diluted in dry N_2_) at 298 K.

Significantly, MFM-100, MFM-101 and MFM-102 retain a degree of structural integrity under low pressures of NH_3_ up to 50 mbar (Fig. S5, ESI†), and it is within this regime that structural determination can be undertaken for NH_3_-loaded MFM-100 (as described above). However, at higher pressures of NH_3_ up to 1 bar loss of crystallinity (lattice periodicity) was observed. This is also evident in the loss or reduction of many of the infrared bands for the framework above 50 mbar (Fig. S11–S13, ESI†). The materials remain amorphous on removal of NH_3_ under vacuum. However, new crystalline MOF materials can be generated from these amorphous materials on desorption of NH_3_ under vacuum for 12 h followed by addition of H_2_O at 298 K for 24 h (Fig. S14, ESI†). This does not re-generate the starting material but another porous crystalline material (denoted R1) with both uptake capacity and crystallinity partially recovered (Fig. 3a and Fig. S15–S17, ESI†).

**Fig. 3:**
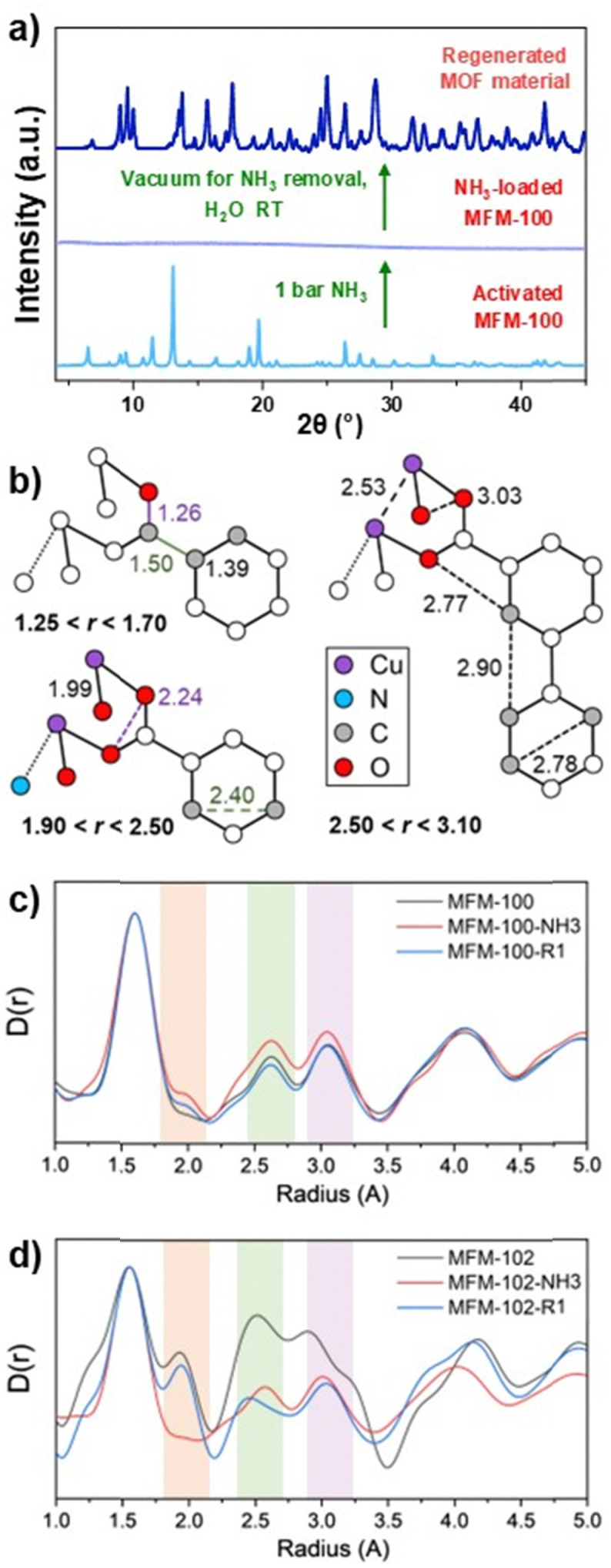
(a) PXRD patterns for as-synthesised and for NH_3_-loaded MFM-100, and for the regenerated crystalline MOF material (R1) formed by placing NH_3_-loaded MFM-100 under vacuum followed by treatment with H_2_O. (b) Structural details of coordination sphere at Cu(ii) in the water-regenerated MOF material: Cu, purple; N, blue; C, grey; O, red. (c) and (d) XPDF pattern of MFM-100 and MFM-102 (bare material), NH_3_-loaded and water regenerated material (R1) in the short-range region with each data set normalized to the most intense peak.

X-ray pair distribution function (XPDF) is an extremely powerful method to determine the local coordination environment and interatomic distances in poorly or non-crystalline materials.^16^ In the short-range region (<5 Å), XPDF patterns afford information on bond formation and cleavage within the first and second coordination sphere, reflected by peak widths and intensities. The long-range region (5–15 Å) carries information of order of periodicity defined by the position of the last significant peak. For bare MFM-100, MFM-101 and MFM-102 long-range peaks are observed, confirming the rigidity and order of the structure. Upon adsorption and desorption of NH_3_ at 298 K and 1 bar, a significant decrease in intensity in these peaks (5–15 Å) was observed for MFM-102 compared to the more structurally rigid MFM-100 (Fig. S18, ESI†). This suggests the loss of long-range periodicity, paralleling the decrease in intensity of Bragg peaks in the PXRD patterns. In contrast, distinctive peaks are observed in the short-range regions (<5 Å) after NH_3_ loading and on regeneration of a new phase on addition of H_2_O. The assignment of peaks below 3.2 Å is illustrated in Fig. 3b. Peaks at 1.8–2.1, 2.4–2.7, and 2.8–3.2 Å are assigned to the Cu–O, Cu–Cu or O–O contacts within the [Cu_2_(O_2_CR)_4_] paddlewheels, respectively. For MFM-100, only small changes in intensity are observed for these peaks on NH_3_ uptake and release, while notable changes of intensity and shift of peak positions were observed for MFM-102 (Fig. 3c and d). A Cu(ii)⋯NH_3_ interaction is observed in MFM-100 and MFM-102 with distances ranging from 2.4 to 2.8 Å, consistent with the SXPD result. The XPDF result suggests that these MOFs undergo relatively small changes in local O-coordination upon adsorption and desorption of NH_3_, but on NH_3_-loading at 1 bar the resultant amorphous MOFs show a lack of long-range order but still retain structural connectivity within the material.

*In situ* EPR spectroscopy was employed to probe the changes to Cu(ii) sites in MFM-100, MFM-101 and MFM-102 during activation, adsorption and regeneration processes. In [Cu_2_(O_2_CR)_4_] paddlewheel structures the two neighbouring Cu(ii), *S* = 1/2 ions couple antiferromagnetically to give an *S* = 0 ground state (EPR silent) and an excited *S* = 1 state (singlet–triplet gap *ca.* 300 cm^−1^), and this is maintained when the paddlewheels are incorporated into MOFs.^15^ The EPR spectra for pristine materials obtained at 150 K at Q- and X-bands (Fig. 4a–d) show characteristic features of the thermally populated *S* = 1 state, spread over a wide field range due to a zero-field splitting (|*D*| *ca.* 0.3 cm^−1^; Tables S2 and S3 and Fig. S19, ESI†). Two further features are observed around the *g* = 2 region (Fig. S19, ESI†): (i) a broad isotropic feature at *g* ≈ 2.1 arising from inter-dinuclear exchange (*J*′ ≈ 1 cm^−1^; Tables S2 and S3, ESI†) between neighbouring *S* = 1 populated [Cu_2_(O_2_CR)_4_] paddlewheels *via* the aryl linker,^17^ and (ii) a sharper, near-axial signal due to monomeric, *S* = ½ Cu(ii) sites. In [Cu_2_(O_2_CR)_4_] paddlewheel MOFs such spectra for monomers have been attributed to extra-framework complexes formed during synthesis. On cooling to 10 K only the isolated Cu(ii) site signals remain as the *S* = 1 state of the paddlewheels is depopulated (Fig. S20, ESI†).^18^ Similar powder patterns have been reported for many materials based on [Cu_2_(O_2_CR)_4_] paddlewheel units.

**Fig. 4:**
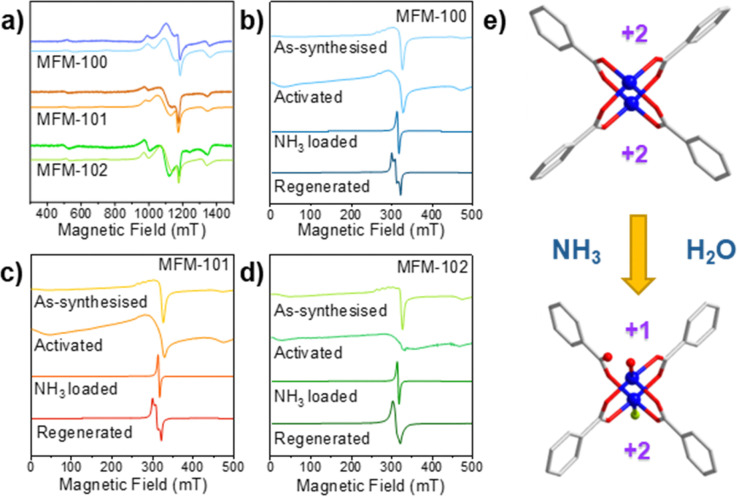
(a) Q-band EPR spectra for MFM-100, MFM-101 and MFM-102 at 150 K (experimental: dark colour; simulated: light colour). (b) to (d) X-band EPR spectra of MFM-100, MFM-101 and MFM-102 at 150 K as-synthesized, activated, NH_3_-loaded and the MOF material regenerated by placing NH_3_-loaded material under vacuum followed by treatment with H_2_O. (e) Schematic representation of coordination at Cu(ii) in activated and regenerated (R1) MOF materials (C, white; O, red; Cu, blue; N, green).

By EPR spectroscopy, the [Cu_2_(O_2_CR)_4_] paddlewheel structures of all the three materials remain intact upon activation (Fig. S21 and S22, ESI†). Adsorption of NH_3_ at 298 K and 1 bar leads to an intense, relatively sharp isotropic signal at *g* = 2.14, observed at both 10 K and 150 K (Fig. 4b–d and Fig. S21 and S22, ESI†). We have been unable to detect a spin echo in pulsed EPR experiments, which suggests rapid electron spin relaxation. This may indicate exchange interactions between Cu(ii) ions, which could be mediated by the hydrogen-bonding network of adsorbed NH_3_ molecules.^19^ No changes in spectra were observed upon desorption of NH_3_ under heating at 100 °C for 2 h, consistent with strongly bound NH_3_ molecules. The spectra of the regenerated R1 materials show a relatively sharp rhombic signal (Fig. 4b–d and Fig. S20–S22, ESI†). The *g*-values (all > *g*_e_; Tables S2 and S3, ESI†) indicate these are associated with Cu(ii), and the lack of Cu hyperfine splitting suggests interaction between sites. We were again unable to detect a spin echo from these signals suggesting that these are due to the framework and not extraneous Cu(ii) sites. The rhombicity suggests a distorted environment, which could indicate induced Cu(ii) defects or ligand displacement after contact with water.^20^ Thus, the rhombicity of the Cu(ii) coordination environment observed here is tentatively assigned to the partial hydrolysis of a Cu–O bond, linked to binding of H_2_O at site I (Fig. 4e). This is also consistent with XPDF results.

In summary, we confirm the adsorption of NH_3_ in a series of Cu(ii)-carboxylate MOFs. MFM-101 shows an NH_3_ uptake of 21.9 mmol g^−1^ at 273 K and 1 bar. Strong Cu(ii)⋯NH_3_ interactions and subtle changes in the coordination geometry of [Cu_2_(O_2_CR)_4_] paddlewheels are observed within a crystalline–amorphous–crystalline phase transition. This work affords insights into NH_3_ adsorption in copper-based porous materials and will inform future development of efficient sorbents for NH_3_.

We thank the EPSRC (EP/I011870, EP/V056409), the Royal Society and the University of Manchester for funding, and the EPSRC for funding of the EPSRC National EPR Facility at Manchester. This project has received funding from the European Research Council (ERC) under the European Union's Horizon 2020 research and innovation programme (grant agreement No 742401, NANOCHEM). We are grateful to the Diamond Light Source for access to beamlines I15-1 and B22. We thank the European Synchrotron Radiation Facility (ESRF) for access to beamline ID22. JC, LG and YM thanks the China Scholarship Council (CSC) for funding.

## Data availability

Data and analyses supporting this article are included in ESI.†

## Conflicts of interest

There are no conflicts to declare.

## Supplementary Material

CC-060-D4CC02604G-s001

CC-060-D4CC02604G-s002

## References

[cit1] Smith C. (2020). et al.. Energy Environ. Sci..

[cit2] Dong B. X. (2016). et al.. Int. J. Hydrogen Energy.

[cit3] Stokstad E. (2014). Science.

[cit4] Rieth A. J. (2019). et al.. Nat. Rev. Mater..

[cit5] Cao D. (2021). et al.. ACS Sustainable Chem. Eng..

[cit6] Qajar A. (2015). et al.. Microporous Mesoporous Mater..

[cit7] Liu J. (2021). et al.. ACS Appl. Mater. Interfaces.

[cit8] Chen Y. (2023). et al.. Acc. Chem. Res..

[cit9] Kim D. W. (2022). et al.. J. Am. Chem. Soc..

[cit10] Han X. (2021). et al.. J. Am. Chem. Soc..

[cit11] Rieth A. J. (2016). et al.. J. Am. Chem. Soc..

[cit12] Snyder B. E. (2023). et al.. Nature.

[cit13] Lin X. (2006). et al.. Angew. Chem., Int. Ed..

[cit14] Peng C. (2017). et al.. J. Environ. Sci..

[cit15] Bleaney B., Bowers K. D. (1952). Proc. R. Soc. London, Ser. A.

[cit16] Firth F. C. (2021). et al.. J. Am. Chem. Soc..

[cit17] Pöppl A. (2008). et al.. J. Phys. Chem. C.

[cit18] Simenas M. (2015). et al.. J. Phys. Chem. C.

[cit19] Borfecchia E. (2012). et al.. J. Phys. Chem. C.

[cit20] Todaro M. (2016). et al.. J. Phys. Chem. C.

