# Supplementary material for: High ammonia adsorption in copper-carboxylate materials: host–guest interactions and crystalline–amorphous–crystalline phase transitions
Source: Chem Commun (Camb). 2024 Sep 16;60(85):12385–8. doi: 10.1039/d4cc02604g (PMC11457036; doi:10.1039/d4cc02604g)
Supplement: CC-060-D4CC02604G-s001 [file CC-060-D4CC02604G-s001.pdf]

Supplementary Information (SI) for ChemComm.
This journal is © The Royal Society of Chemistry 2024

Supporting Information

High ammonia adsorption in copper-carboxylate materials: host-guest interactions and crystalline-amorphous-crystalline phase transitions

Wanpeng Lu,[1] Yinlin Chen,[1] Zi Wang,[1] Lixia Guo,[2] Jin Chen,[1] Yujie Ma,[1] Weiyao Li,[1] Jiangnan Li,[2] Meng He,[1] Mengtian Fan,[1] Alena M. Sheveleva,[1,3] Floriana Tuna,[1,3] Eric J. L. McInnes,[1,3] Mark D. Frogley,[4] Philip A. Chater,[4] Catherine Dejoie,[5] Martin Schröder[1]* and Sihai Yang[1,2]*

1. Department of Chemistry, University of Manchester, Manchester, M13 9PL, UK
2. Beijing National Laboratory for Molecular Sciences, College of Chemistry and Molecular Engineering, Peking University, Beijing 100871, China
3. Photon Science Institute, University of Manchester, Manchester, M13 9PL, UK
4. Diamond Light Source, Harwell Science Campus, Oxfordshire, OX11 0DE, UK
5. European Synchrotron Radiation Facility, Grenoble, 38043, France

List of Contents

1. Experimental Section
2. Structures and Adsorption Isotherms
3. Cycling Experiments and Calculation of Q_{st}
4. Thermal Programmed Desorption Measurements and UV-vis Spectroscopy
5. *In situ* Synchrotron Powder X-ray Diffraction
6. Single Crystal Synchrotron FTIR Micro-spectroscopy
7. Materials Regeneration and Characterization
8. X-ray Pair Distribution Function Analysis
9. Electron Paramagnetic Resonance (EPR) Spectroscopy
10. X-ray Photoelectron Spectroscopy
11. Comparison of Ammonia Adsorption Capacity in MOFs

1. Experimental Section

1.1 Synthesis of the MOF Materials and General Characterization

All the reagents were used as received from commercial suppliers without further purification. Syntheses of MFM-100, MFM-101, MFM-102, MFM-126, MFM-127, MFM-190(F), MFM-170, Cu-MOP-1a were carried out using previously reported methods.[1–5] Powder X-ray diffraction (PXRD) patterns were collected using a Philips X'pert X-ray diffractometer (40kV and 30 mA) using Cu-Kα radiation (λ = 1.5406 Å). Ultraviolet-visible (UV-vis) spectra were recorded on a UV-vis spectrophotometer (Shimadzu, UV 2600). Elemental analyses were performed using a Thermo Scientific iCAP 6000 Series ICP spectrometer and a Thermo Scientific Flash 2000 organic elemental analyser. N_2 adsorption isotherms were measured on a Tristar II PLUS (Micrometrics) instrument at 77 K, and XPS spectra were measured using a Kratos Axis Ultra instrument equipped with a monochromatic Al ka X-ray source (E = 1486.6 eV). SEM (scanning electron microscopy) images were obtained with an Hitachi SU8000 cold field emission Scanning Electron Microscope.

1.2 Adsorption Isotherm and Cycling Experiment

Measurements of static adsorption isotherms (0–1.0 bar) for NH_3 were carried out using an IGA gravimetric sorption analyser (Hiden Isochema, Warrington, UK). Desolvated (activated) samples of MFM-100, MFM-101, MFM-102, MFM-126, MFM-127, MFM-190(F), MFM-170, Cu-MOP-1a were generated *in situ* under dynamic vacuum (1×10^{-8} mbar) by heating at 373 K for 12 h. Research grade NH_3 was purchased from BOC and used as received. For cycling experiments, the pressure of NH_3 was increased from vacuum (1×10^{-8} mbar) to 150 mbar and the uptake recorded. The pressure was then reduced to regenerate the sample with no assisted heating and the uptake recorded. This cycling process was repeated for 5 cycles and the difference between adsorption and desorption uptake calculated.

1.3 Isosteric enthalpy and entropy calculation

The isosteric enthalpy of adsorption (ΔH_n below) and entropies (ΔS_n) for NH_3 uptake are calculated as a function of loading (n), all isotherms (273 K to 303 K) were fitted to the van't Hoff isochore:

$$ln(p)_n = \frac{\Delta H_n}{RT} - \frac{\Delta S_n}{R}$$

A plot of ln*(p)* versus 1/*T* at constant loading allows the differential enthalpy and entropy of adsorption and the isosteric enthalpy of adsorption (Q_{st}, n) to be determined.

1.4 Breakthrough and Thermal Programmed Desorption Measurement

Breakthrough experiments were performed on Hiden Isochema IGA-003 with ABR attachments and mass spectrometer for gas detection. The MOF samples were activated at 373 K under dynamic vacuum for 12 h before breakthrough experiments, and 600 mg of desolvated MOF sample was packed into the fixed-bed reactor. The sample was then heated at 373 K under He flow for 2 h to achieve further activation. The fixed-bed was then cooled to 298 K and the breakthrough experiment performed with a stream of 1000 ppm NH_3 (diluted in He). The flow rate of the gas mixture was 15 mL min^{-1}. The gas concentration, C, of NH_3 at the

outlet was compared with the corresponding inlet concentration C_0, where $C/C_0 = 1$ indicates complete breakthrough.[6] NH_3-TPD experiments were carried out to test the NH_3 binding in the MOF materials. Typically, the activated sample was treated with a stream of 1000 ppm NH_3 diluted in He. A Bruker Matrix MG5 FTIR spectrometer was used to analyse the outlet gases for the detection of NH_3, H_2O and CO_2. The Bruker MATRIX-MG5 features a 5 m multi-reflection gas cell and is designed for the high-precision quantification of gas compounds from very low concentrations on the ppb level up to one hundred percent. The gas analysis system uses the certain sections of FTIR spectrum that are unique to a given gas to first identify the gas is present and then uses a fitting algorithm to quantity the amount of gas present. If other gases interfere with the signal, then the system also fits these interfering gases and takes them into account when performing the quantification. The system was calibrated and set up before use. When the outlet concentration of NH_3 was equal to that of the inlet, 100 mL min^{-1} of pure He was used to flush the sample for about 2 h, removing the physically adsorbed NH_3. The NH_3-TPD experiments were then carried out at a heating rate of 5 °C min^{-1} from 30 °C to 300 °C.

1.5 *In situ* Synchrotron X-ray Powder Diffraction

Synchrotron powder X-ray diffraction was carried out on the ID22 high-resolution powder diffraction beamline at the European Synchrotron Radiation Facility (ESRF). Data were collected between 0 and 35° with a 13 channel multianalyser stage under the wavelength of 0.354267(4) Å. Data were binned using a step size of 0.002°. A freshly synthesised sample of MFM-100, MFM-101, and MFM-102 was exchanged with acetone over the course of 1 week before being dried under dynamic vacuum and loaded into a 0.7 mm borosilicate capillary. The loaded capillary was activated *in situ* by heating to 100 °C under active vacuum for 2 h using a temperature-controlled Oxford Cryosystems open-flow N_2 gas cryostat. Activation of the sample was visualised by a colour change from light blue to purple and diffraction data was collected. 5% NH_3 in He was flowed into the capillary and diffraction data was collected after 1 h stabilization.

Rietveld refinement of the structure was carried out on data between 1 and 25° using the TOPAS software package. Atomic parameters for the reported structure of MFM-100 (CCDC no. 257470) were used as a starting point before adding successive NH_3 molecules (with hydrogens omitted) into the framework. Approximate positions for NH_3 molecules were found using the simulated annealing approach before further refinement to find the optimal orientation of the guest molecules. Accuracy of the final model was verified by the convergence of the weighted profile factor (Rwp), the chemical sense of the model and the good correlation between the observed and calculated diffraction patterns. Crystal data of MFM-100_NH_3 are deposited at Cambridge Crystallographic Data Centre (CCDC) 2175724.

1.6 Single Crystal Synchrotron FTIR Micro-spectroscopy

In situ gas-loaded single crystal synchrotron FTIR micro-spectroscopy was carried out at the Multimode InfraRed Imaging and Microspectroscopy (MIRIAM) beamline at the Diamond Light Source, Harwell Science Campus (UK). The instrument is comprised of a Bruker Hyperion 3000 microscope in transmission mode with

a 15× objective and condenser, and a liquid N_2 cooled MCT detector (mid-band, 50 um element), coupled to a Bruker Vertex 80V Fourier Transform IR interferometer using radiation generated from a bending magnet source. Spectra were collected (512 scans) in the range 500–4000 cm^{-1} at 4 cm^{-1} resolution and infrared spot size at the sample of approximately 30 × 30 μm. Samples were placed onto a zinc selenide (ZnSe) disk and placed within a Linkam FTIR 600 gas-tight sample cell, which was equipped with ZnSe windows, a heating stage and gas inlet and outlets.

NH_3 was dosed volumetrically into the sample cell using mass flow controllers, and the total flow rate was maintained at 100 cm^3 min^{-1} for all experiments. The gases were directly vented to an exhaust system and the total pressure in the cell was maintained at 1 bar for all experiments. The single crystal samples of MFM-100, MFM-101 and MFM-102 were desolvated under a flow of dry N_2 at 100 cm^3 min^{-1} and 373 K for 2 h, and then cooled to room temperature under a continuous flow of N_2. For all single crystal samples, the initial gas flow was pure N_2 at a flow rate 100 cm^3 min^{-1}, which was then switched to 1%, 2%, 5%, 10%, 20%, 40%, 60% of NH_3 diluted in dry N_2.

1.7 Materials regeneration

The regenerated materials were obtained after contact with 1 bar NH_3 or after running the NH_3 isotherms at 1 bar. The physically adsorbed NH_3 was removed by placing loaded material under dynamic vacuum (1×10^{-8} mbar) for 1 h. The obtained MOF was placed in deionised water for 24 h and suspended by sonication for 0.5 hour. The mixture was then filtered and exchanged with acetone to afford the regenerated material.

1.8 X-ray Pair Distribution Function Analysis

XPDF patterns were collected at beamline I15- at Diamond Light Source with an X-ray wavelength λ = 0.189578 Å. Initial calibration measurements were performed on an empty 1 mm borosilicate capillary. Desolvated MFM-100, MFM-101 and MFM-102 were prepared by heating the sample at 100 °C under dynamic vacuum of 10^{-7} mbar. Research grade NH_3 was purchased from BOC and used as received for NH_3 adsorption. The samples after dosing of NH_3 were evacuated under 10^{-7} dynamic vacuum for 2 h and soaked in H_2O for to afford the regenerated materials. The samples were then ground and loaded into 1 mm borosilicate capillaries for data collection. The data were processed using GudrunX[7] and PDFGui[8] software.

1.9 Electron Paramagnetic Resonance Spectroscopy

For electron paramagnetic resonance (EPR) studies, the tube size and tube position in the cavity were kept constant, and Q-band samples prepared in 1.6 mm capillaries for measurements. Desolvated MFM-100, MFM-101 and MFM-102 were prepared by heating the sample at 100 °C in a J. Young X-band EPR tube (4 mm) at 10^{-7} mbar. For NH_3 adsorption studies, the sample in a J. Young X-band EPR tube was dosed with NH_3. The samples after dosing with NH_3 were evacuated under 10^{-7} dynamic vacuum for 2 h, loaded with H_2O and kept under room temperature for 24 h in J. Young X-band EPR tubes. Low temperature CW EPR spectra (10 K and 150 K) were measured with a Bruker EMX 300 EPR spectrometer equipped with X-band (ca. 9.4 GHz) and

Q-band (ca. 35 GHz) resonator and a liquid He cryostat. Field corrections were applied by measuring relevant EPR standards (Bruker Strong Pitch). Simulation of the EPR spectra was performed with the EasySpin/MATLAB toolbox, which employs the exact diagonalization of the spin Hamiltonian matrix.[9]

2. Structures and Adsorption Isotherms

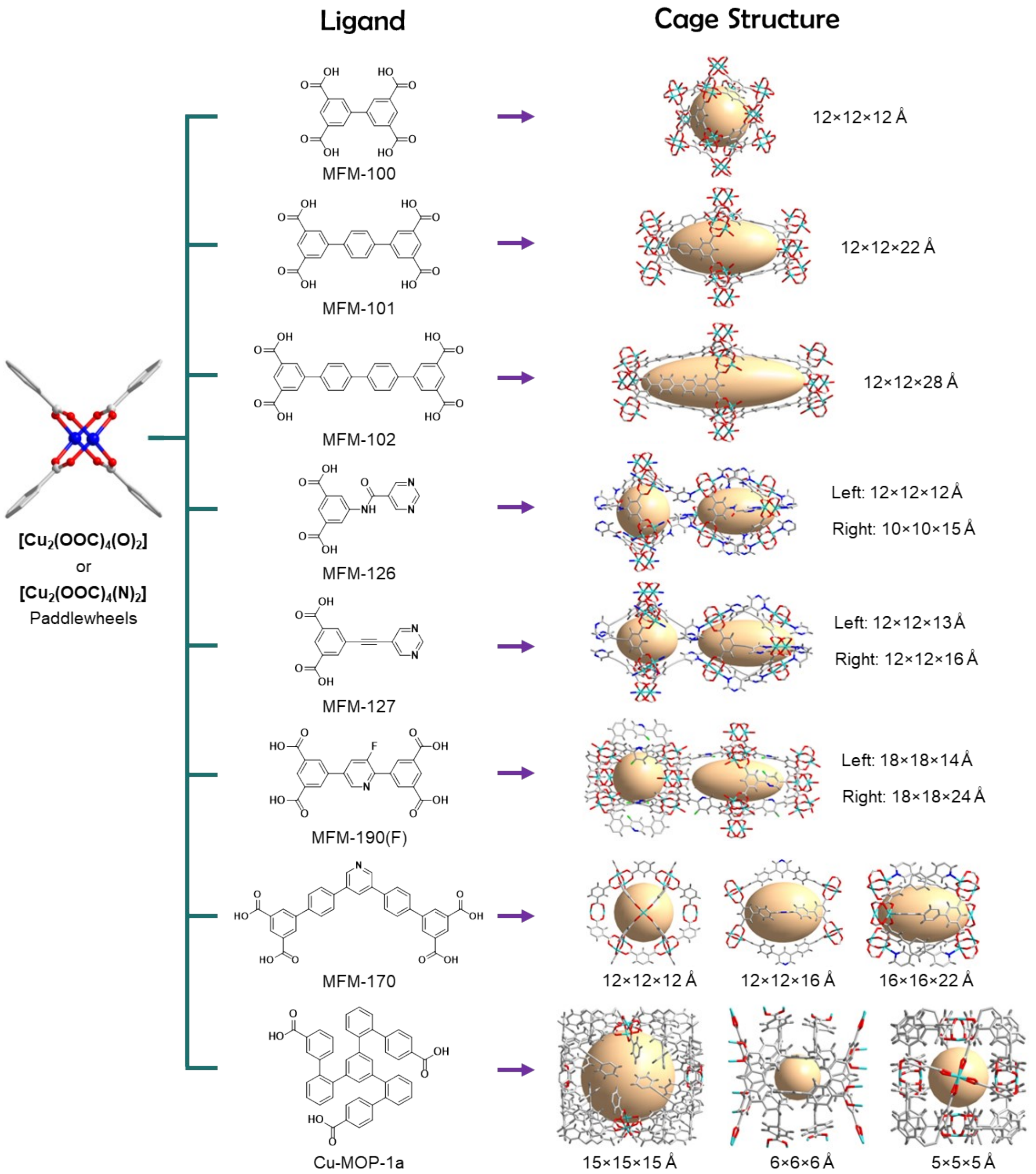

Figure S1. Views of ligands and crystal structures of the MOF/MOP materials in this study.

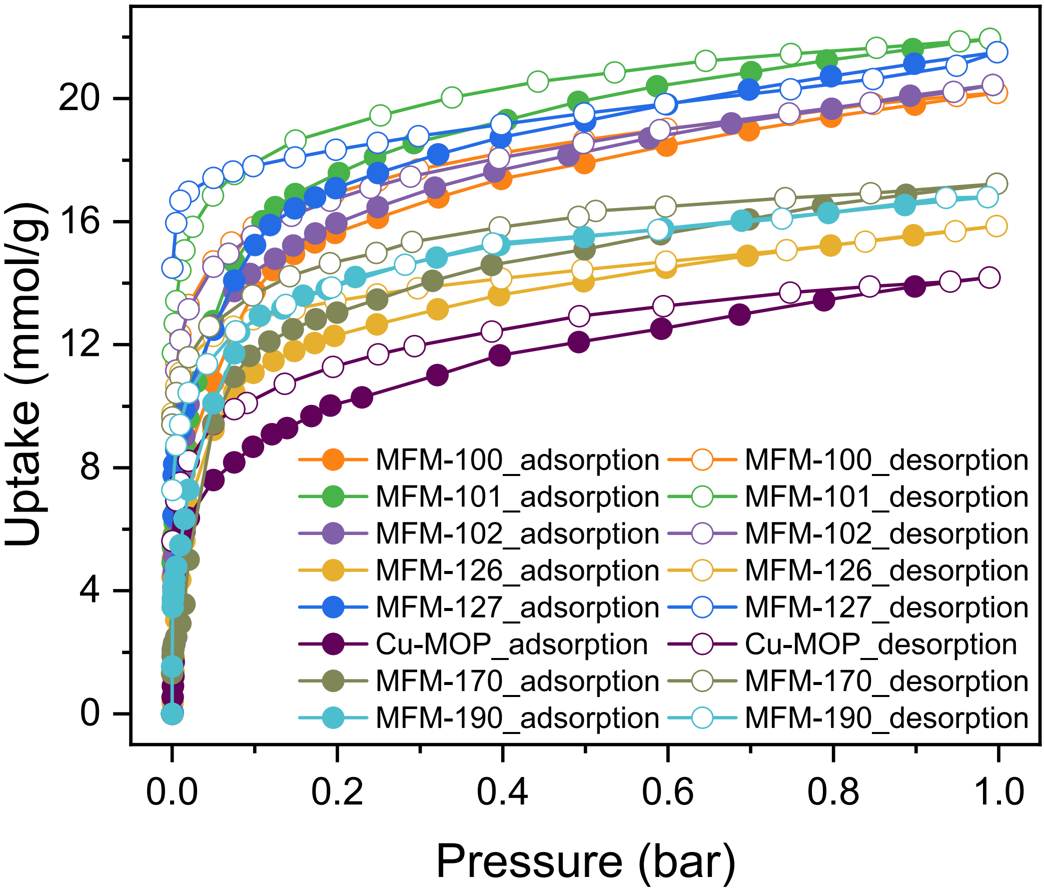

Figure S2. Adsorption-desorption isotherms for NH_3 at 273 K up to 1 bar.

Table S1 Summary of ammonia adsorption of the materials in this study.

MOF	NH_3 Uptake (273K 1bar) /mmol g^{-1}	BET /m^2 g^{-1}	Pore Volume (Single Crystal) /cm^3 g^{-1}	Metal Centre Density /nm^3	NH_3 Packing density in MOF pores/ g cm^{-3}	NH_3 storage density in MOFs /g cm^{-3}
MFM-100	19.8	1108	0.68	2.44	0.58	0.33
MFM-101	21.9	1722	1.08	1.55	0.63	0.33
MFM-102	20.4	2404	1.28	1.17	0.52	0.35
MFM-126	15.9	1004	0.52	1.74	0.75	0.36
MFM-127	21.5	1557	0.57	1.78	0.70	0.37
Cu-MOP-1a	14.2	751	0.38	1.23	0.79	0.25
MFM-170	17.2	2408	0.87	1.27	0.34	0.21
MFM-190(F)	16.8	2537	0.83	1.56	0.35	0.22

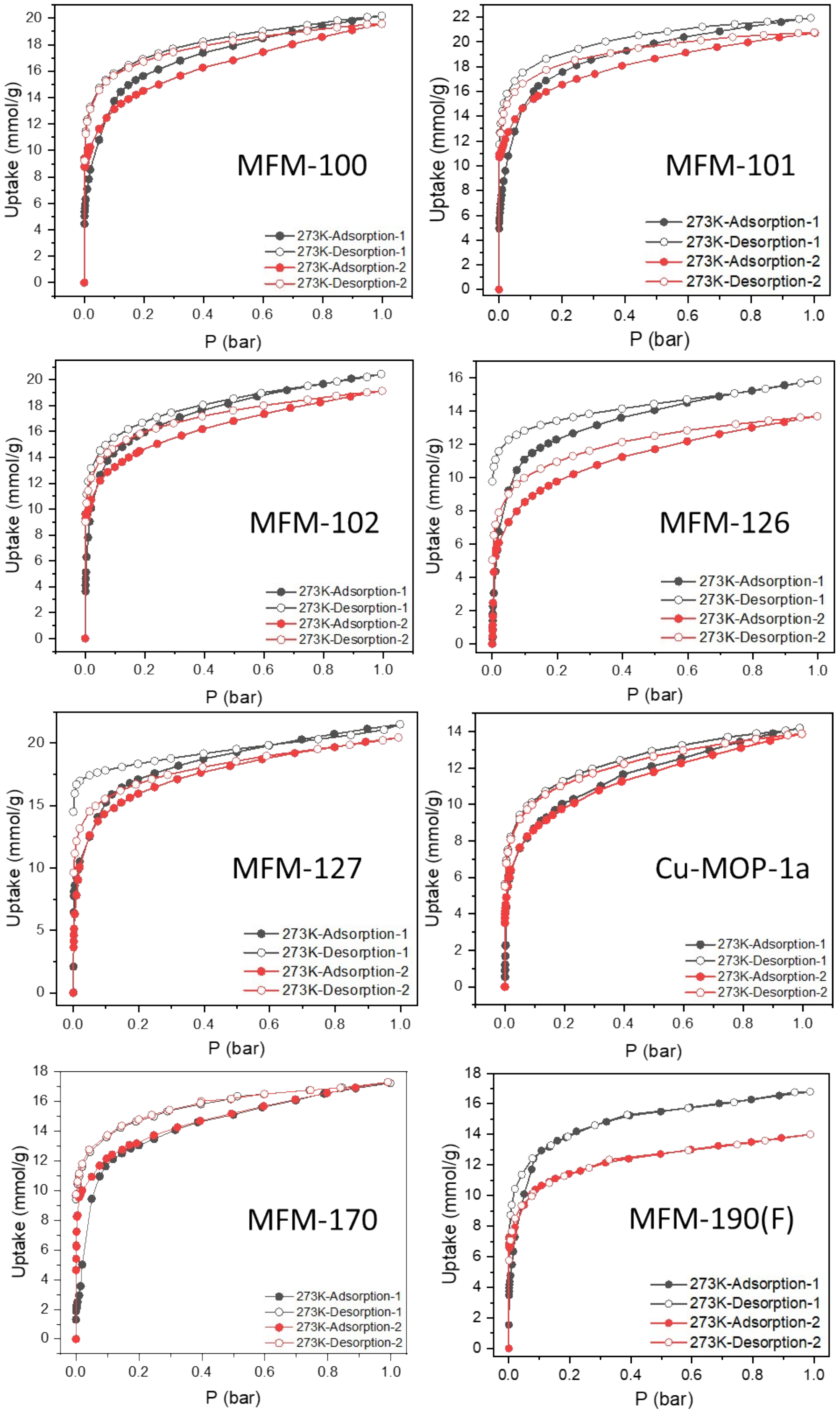

Figure S3. Adsorption-desorption isotherms for NH_3 at 273 K up to 1 bar in the materials studied herein.

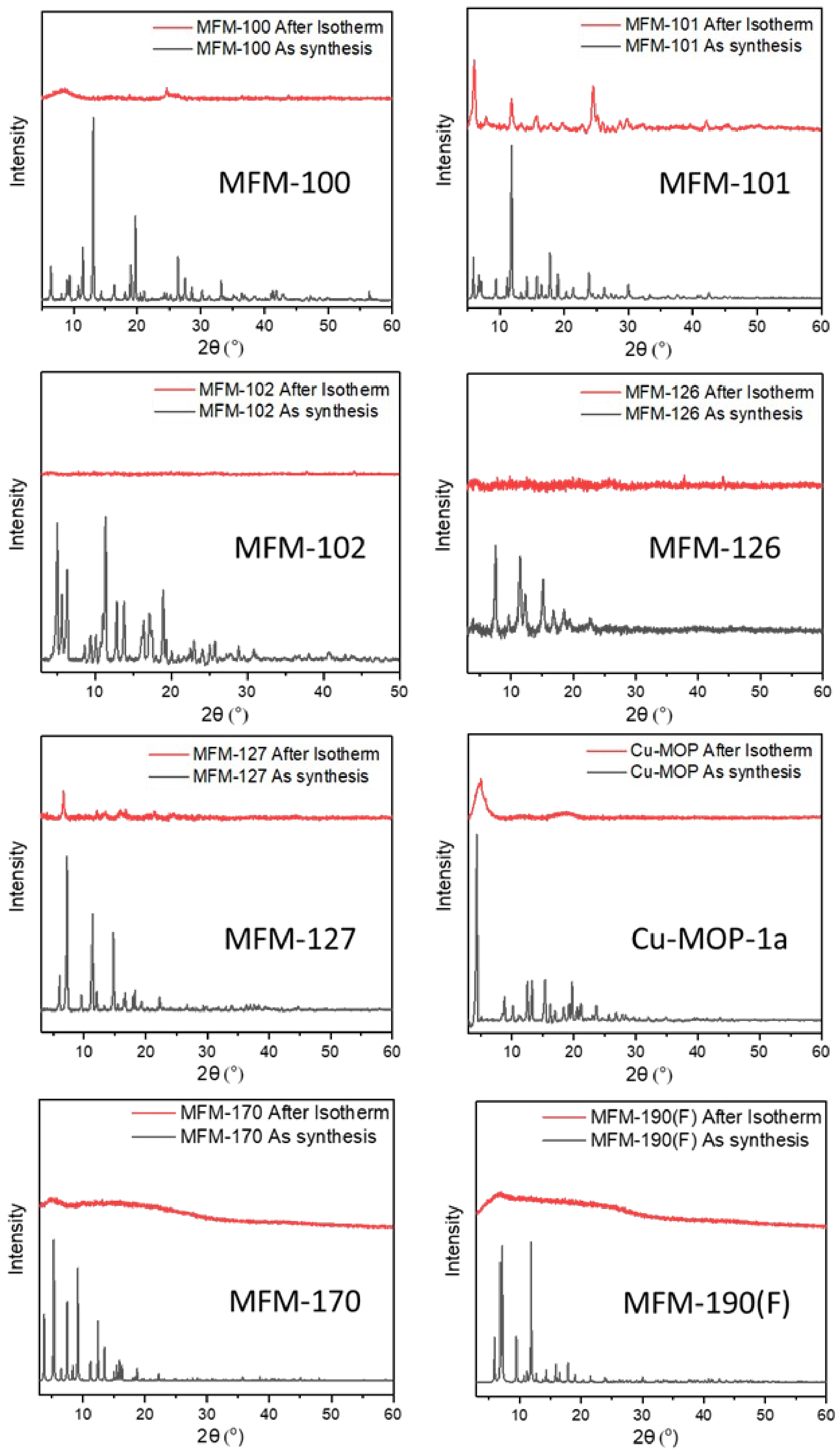

Figure S4. PXRD pattern for materials before and after NH_3 isotherms at 273 K up to 1 bar.

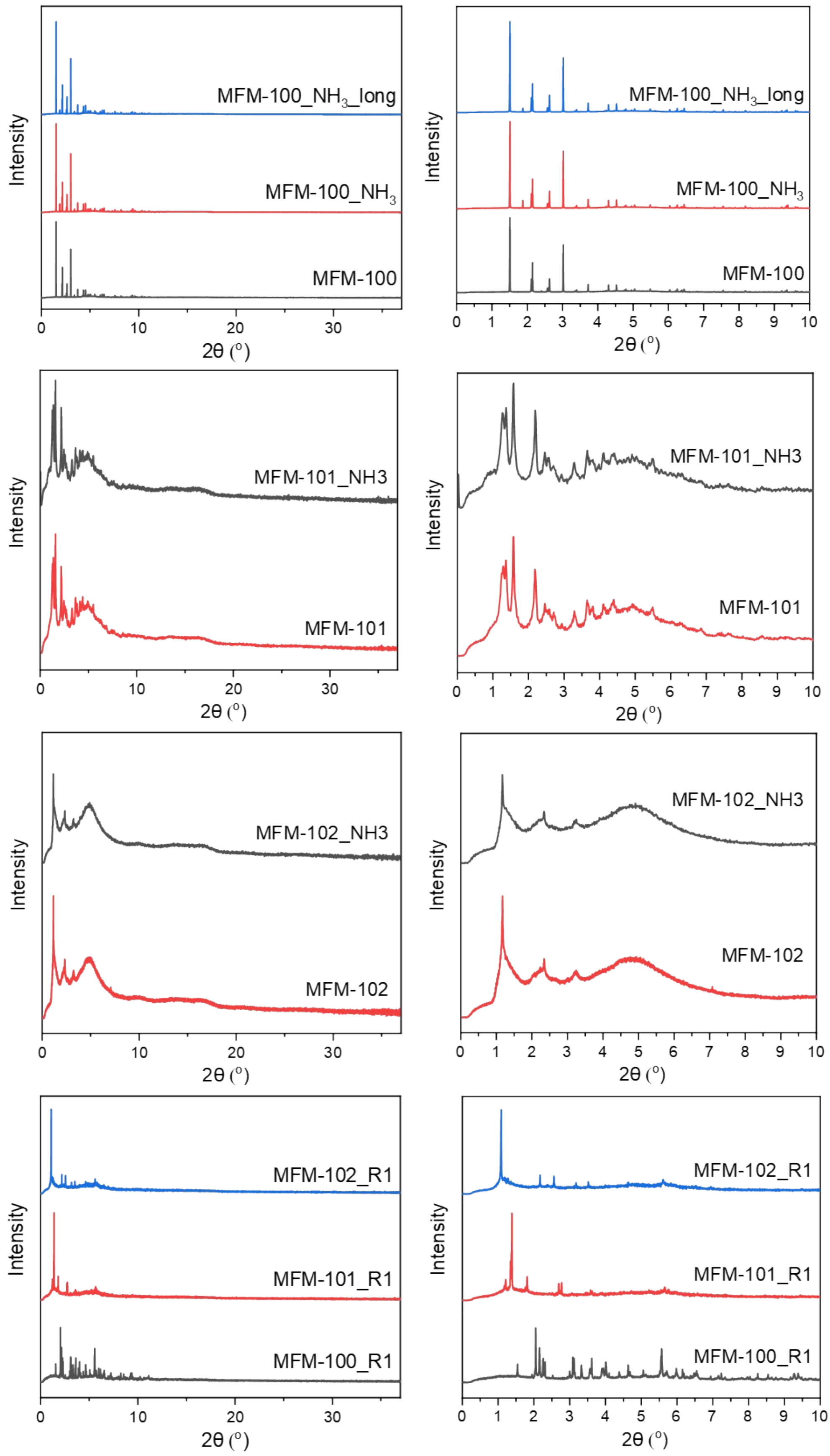

Figure S5. Synchrotron PXRD patterns of MFM-100, MFM-101 and MFM-102: as synthesised, after adsorption of NH_3 (5%) and regenerated (R1) materials (left: 2θ before 37°; right: 2θ before 10°; _long: long term exposure to low concentration NH_3 after 2 hours).

3. Cycling Experiments and Calculation of Q_{st}

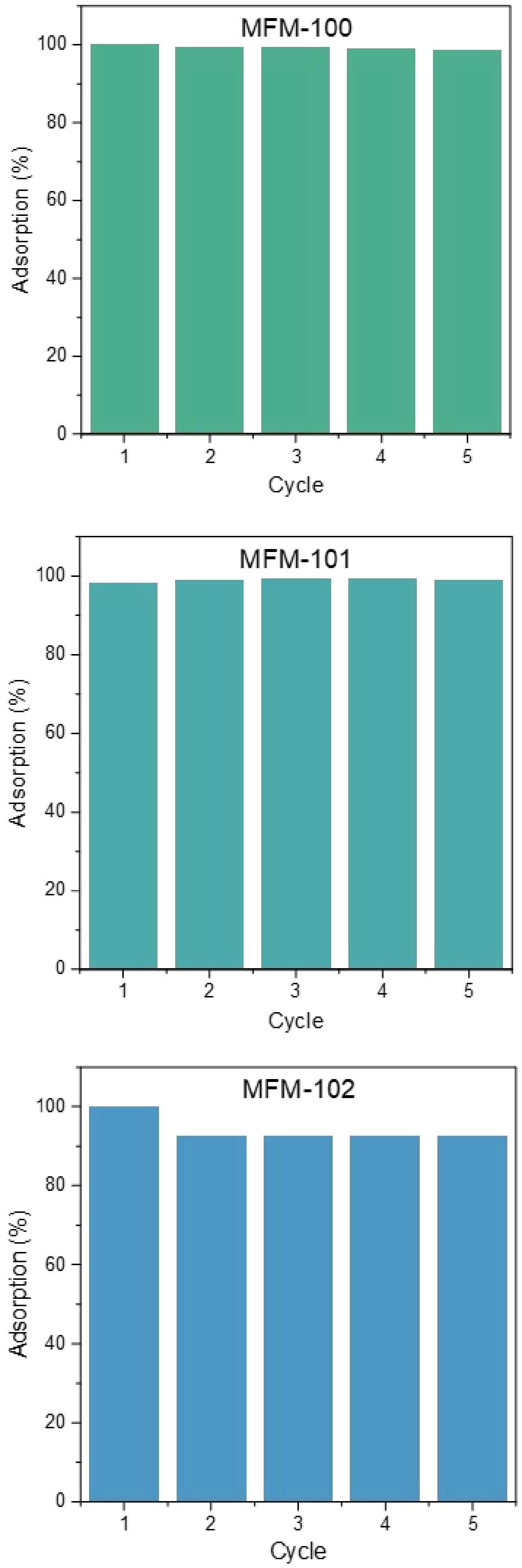

Figure S6. Cycling performance of MFM-100, MFM-101 and MFM-102 for uptake of NH_3 at 298 K between 0 and 0.15 bar.

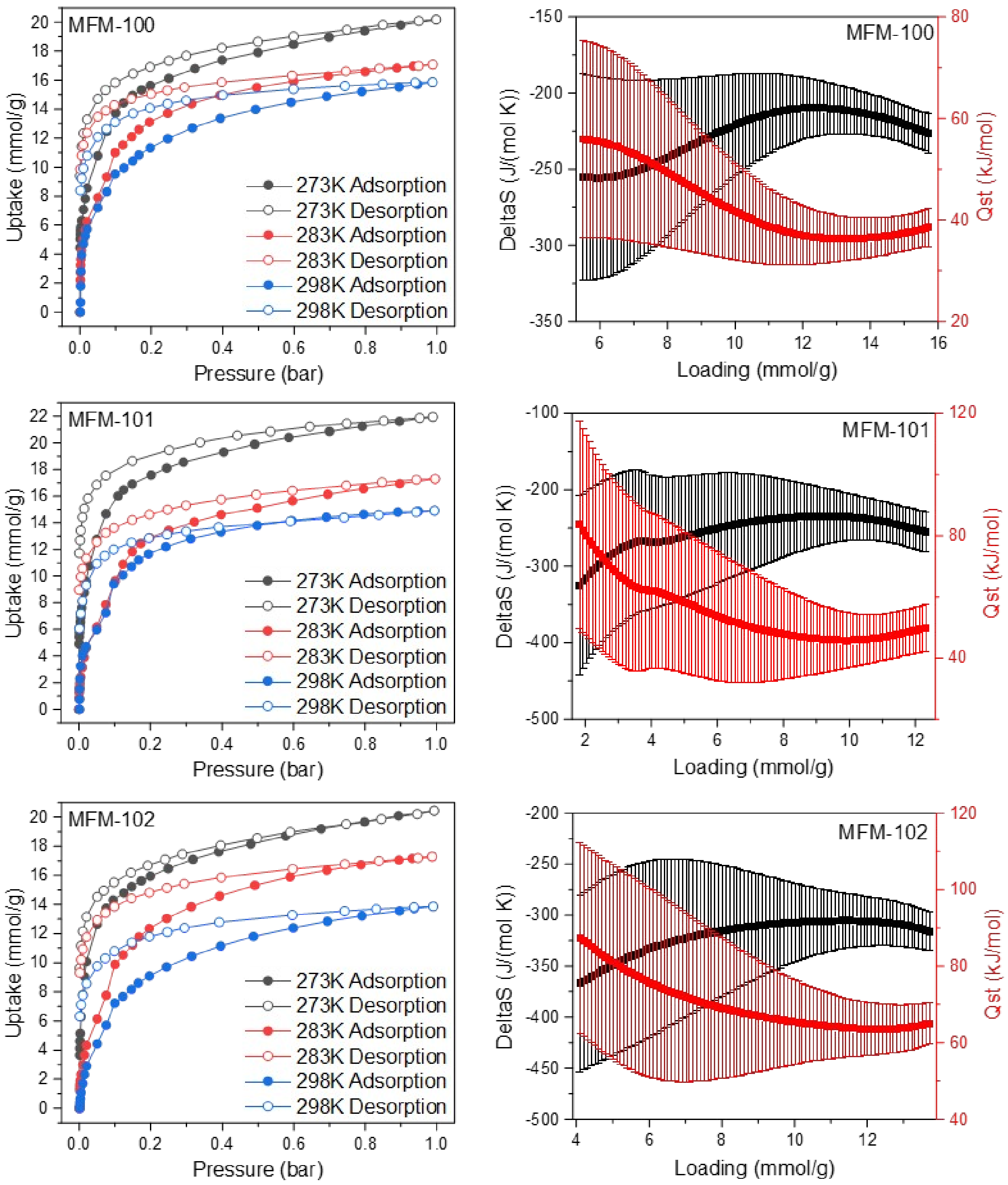

Figure S7. Isotherms and Q_{st} plots for MFM-100, MFM-101 and MFM-102.

4. Thermal Programmed Desorption Measurements and UV-vis Spectroscopy

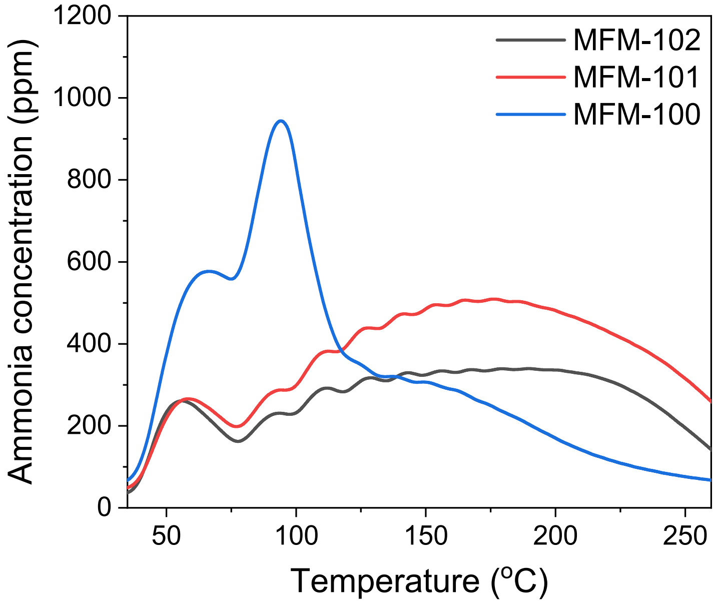

Figure S8. Temperature-programmed desorption (TPD) of NH_3 in MFM-100, MFM-101 and MFM-102, showing variation of NH_3 concentration in the outlet gas stream as a function of temperature.

5. *In situ* Synchrotron Powder X-ray diffraction

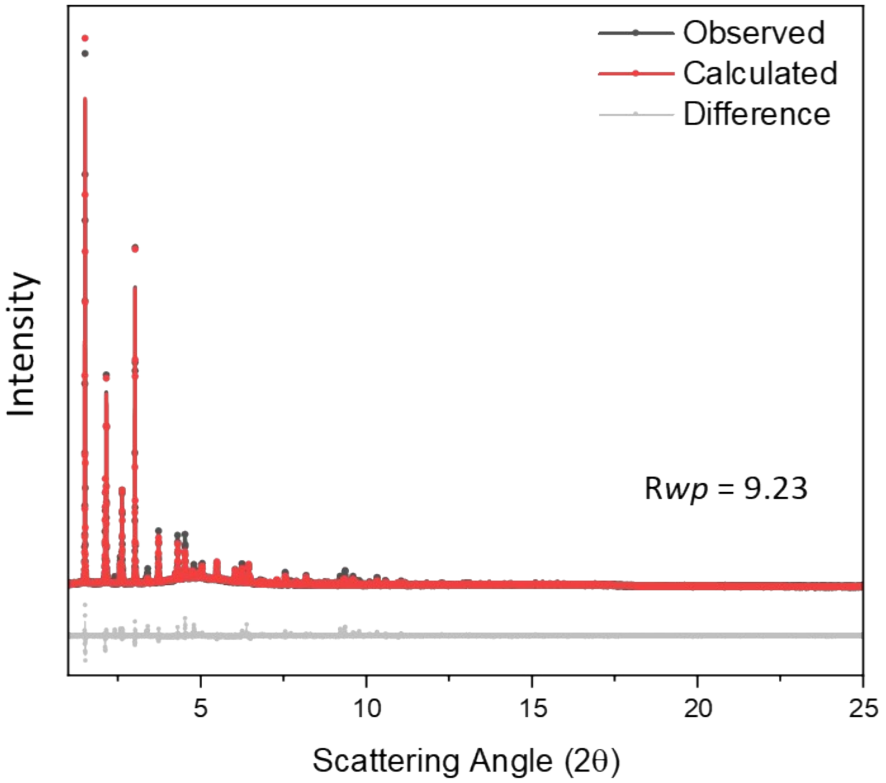

Figure S9. Synchrotron PXRD pattern and Rietveld refinement for MFM-100. R*wp* = 9.23.

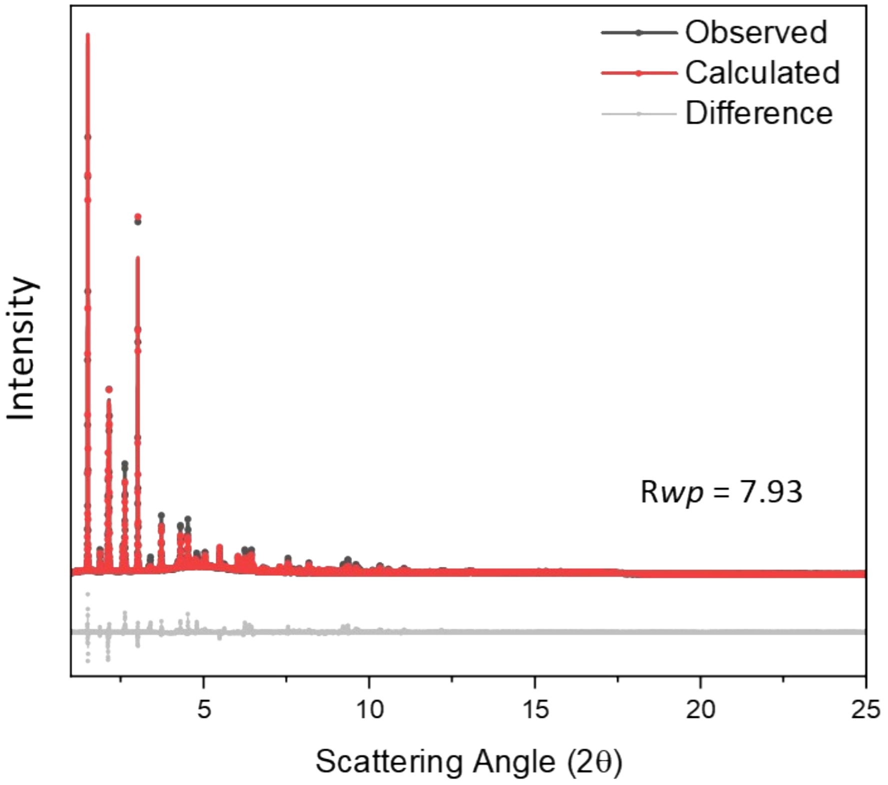

Figure S10. Synchrotron PXRD pattern and Rietveld refinement for MFM-100·4.4NH_3. R*wp* = 7.93.

6. Single Crystal Synchrotron FTIR Micro-spectroscopy

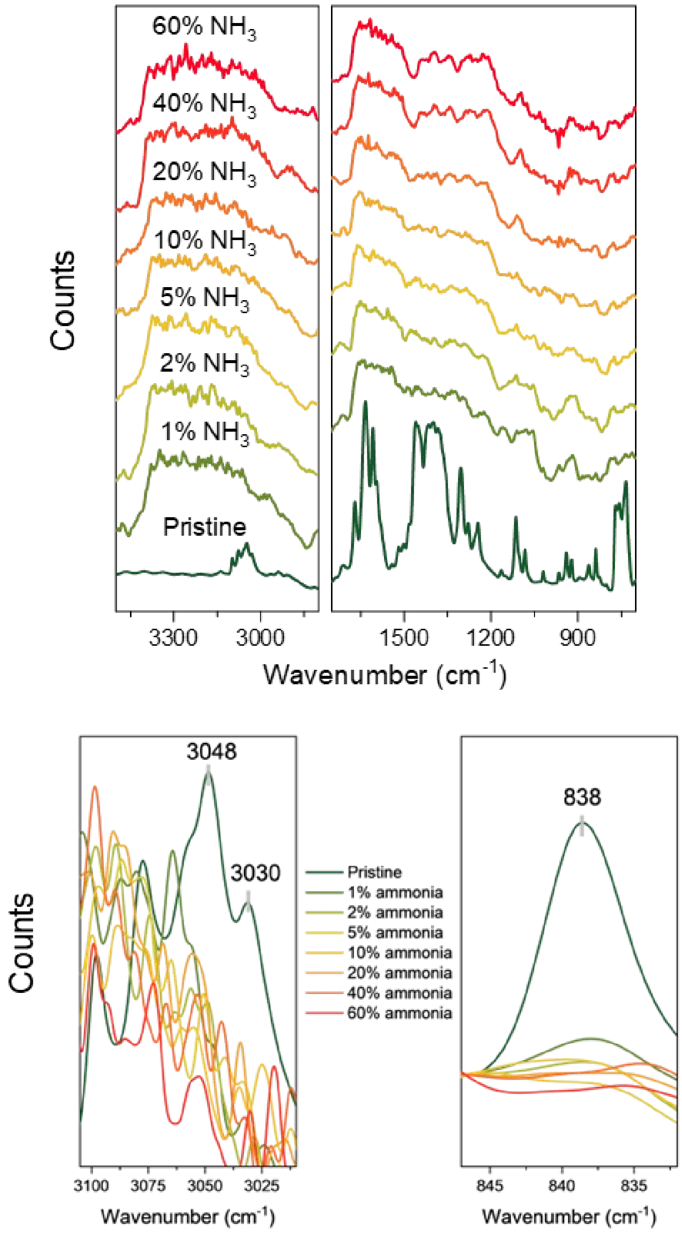

Figure S11. *In situ* synchrotron FTIR spectra for single crystals of MFM-100 under various concentrations of NH_3 diluted in dry N_2 (bottom left: aromatic C–H stretching; bottom right: aromatic C–H bending).

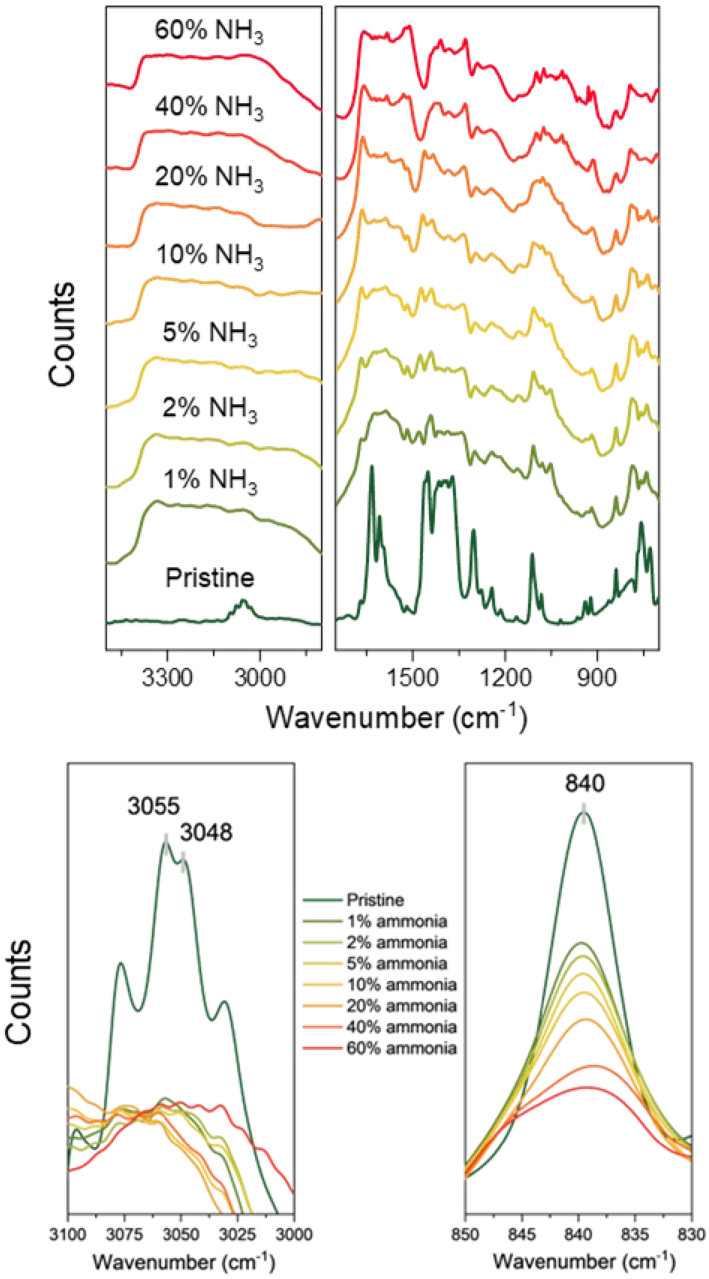

Figure S12. *In situ* synchrotron FTIR spectra for single crystals of MFM-101 under various concentrations of NH_3 diluted in dry N_2 (bottom left: aromatic C–H stretching; bottom right: aromatic C–H bending).

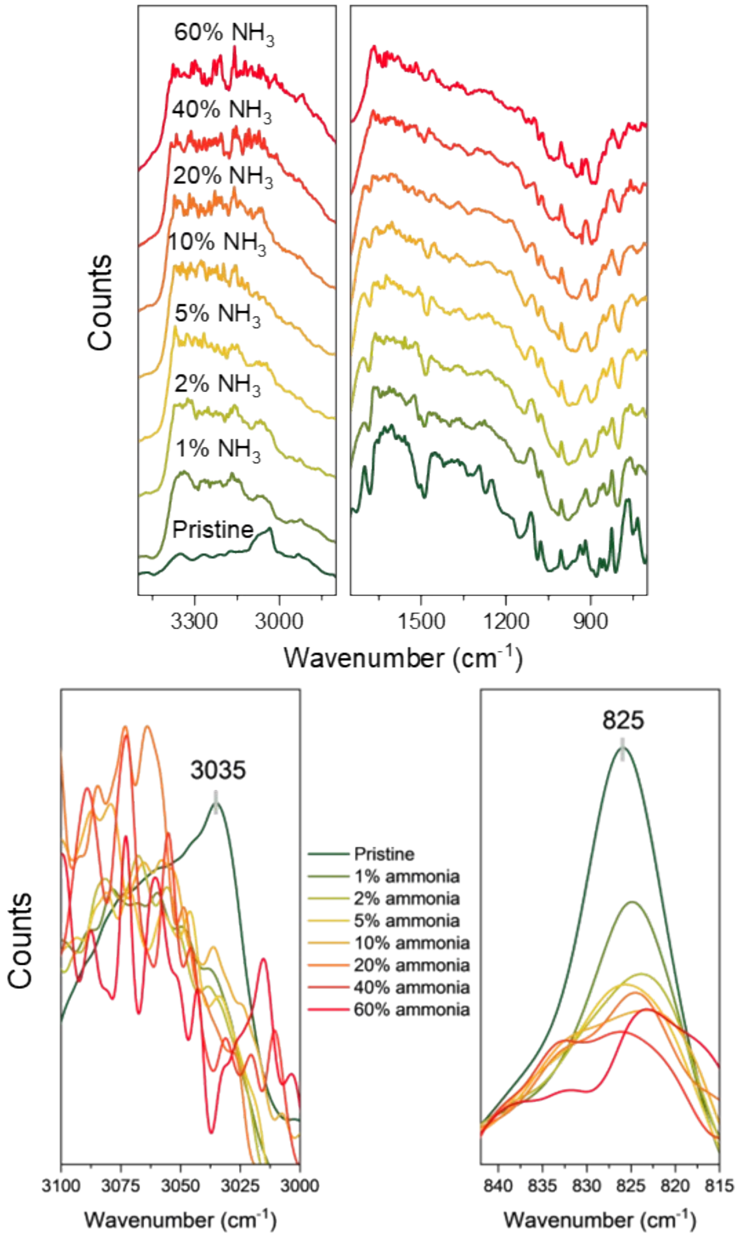

Figure S13. *In situ* synchrotron FTIR spectra for single crystals of MFM-102 under various concentrations of NH_3 diluted in dry N_2 (bottom left: aromatic C–H stretching; bottom right: aromatic C–H bending).

7. Material regeneration and characterization

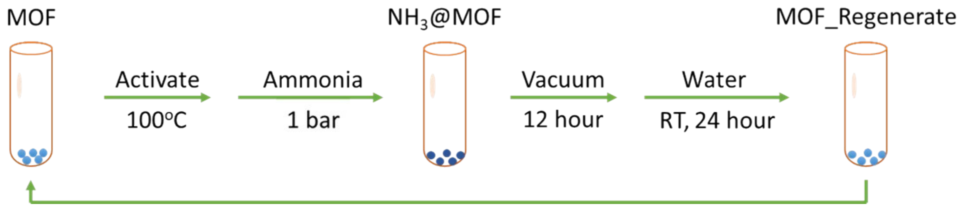

Figure S14. Scheme for the regeneration of MOF materials.

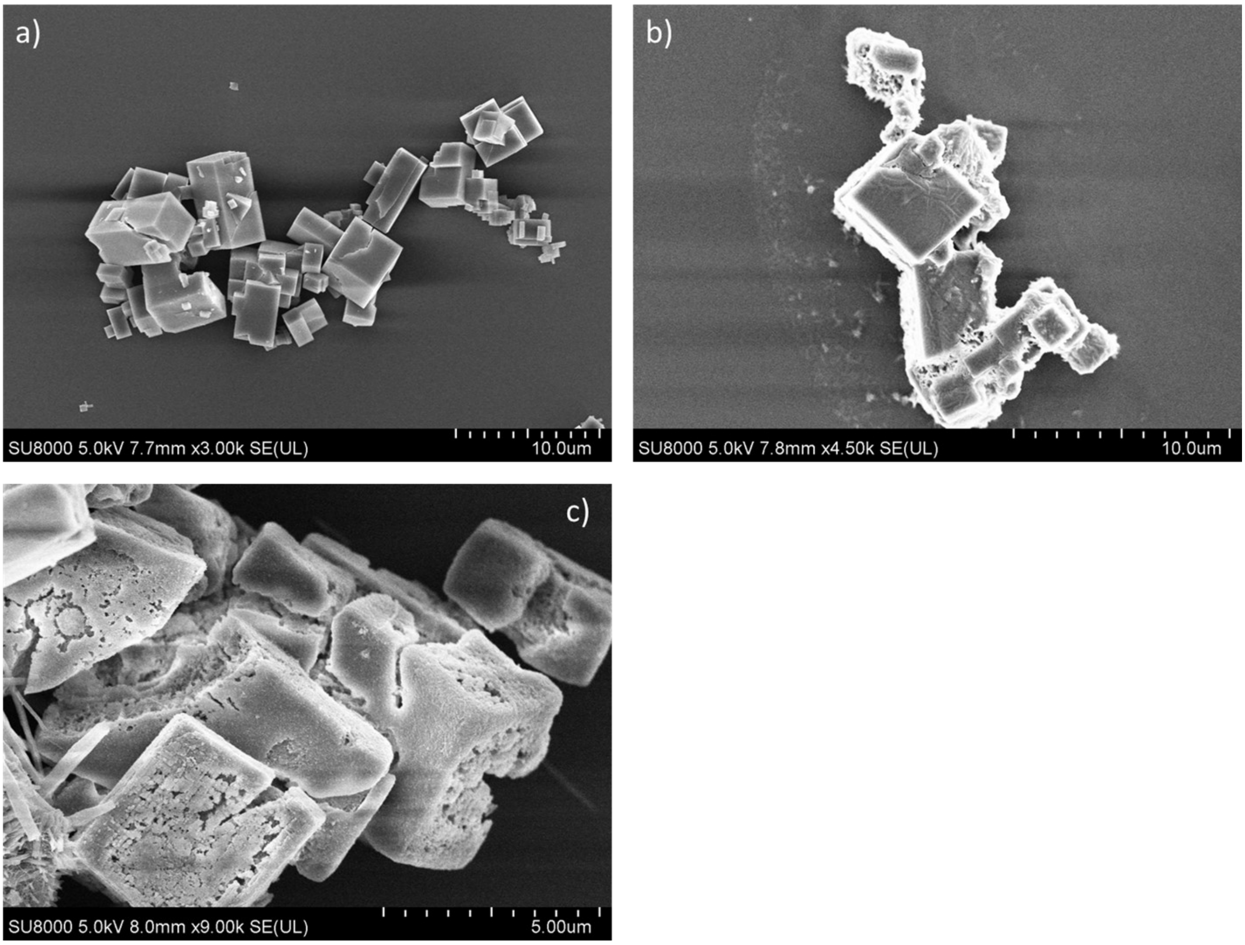

Figure S15. SEM images for a) MFM-100-as synthesised, b) MFM-100-NH_3 loaded and c) MFM-100-regenrated.

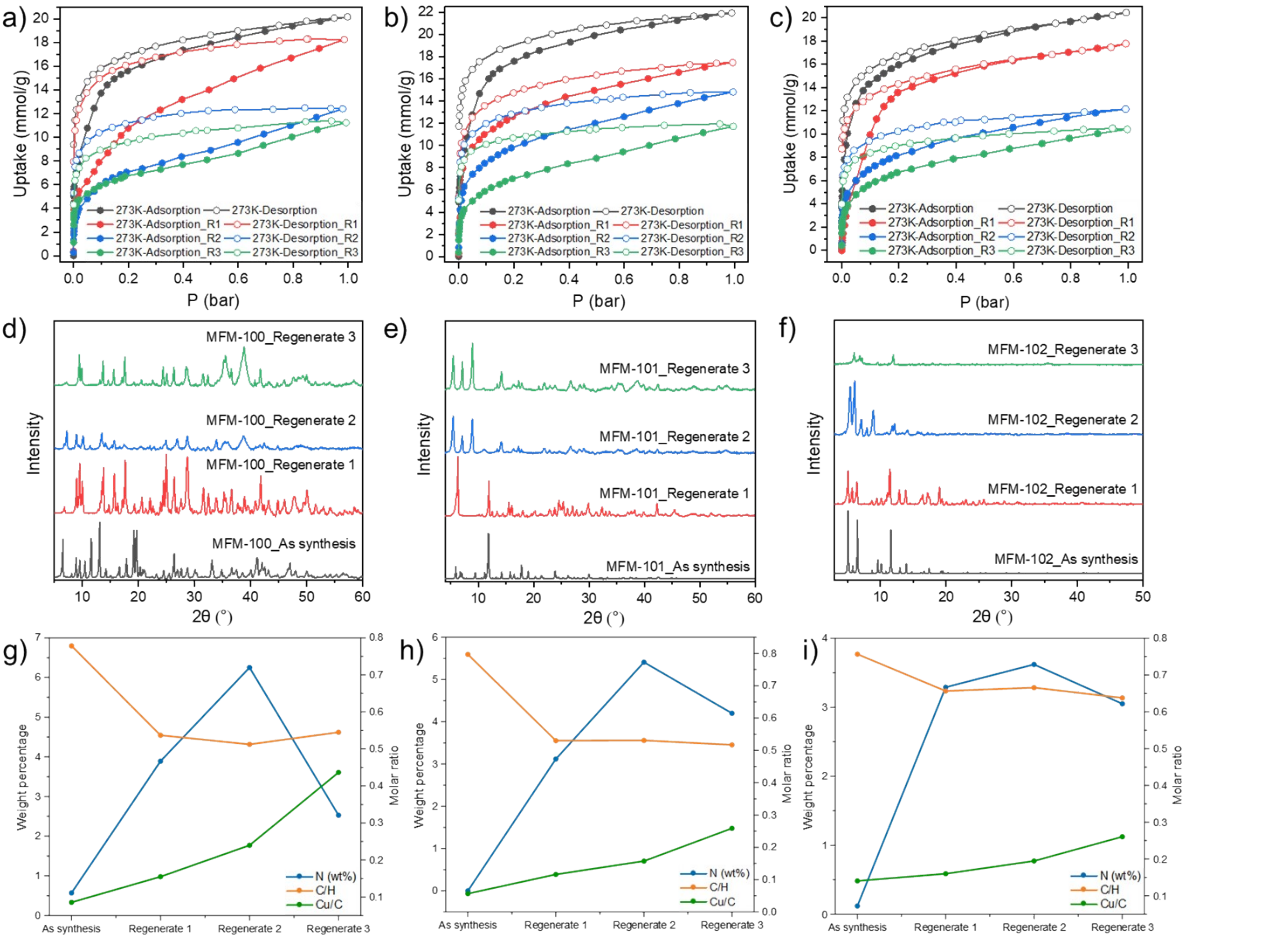

Figure S16. a-c) NH_3 isotherms of regenerated materials at 273K. d-f) PXRD patterns for regenerated materials. g-i) Elemental analysis of regenerated materials.

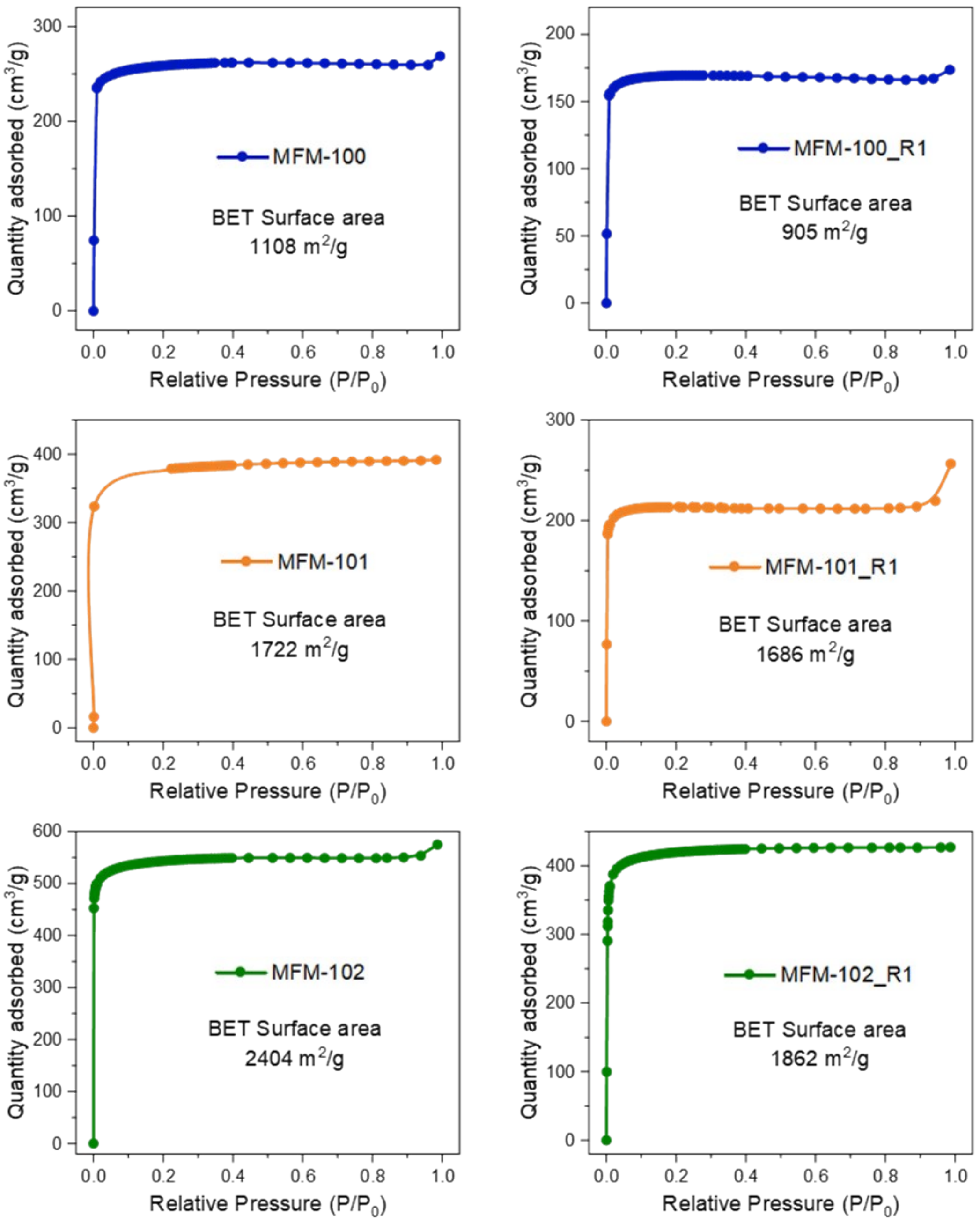

Figure S17. BET surface areas of MFM-100, MFM-101 and MFM-102 derived from N_2 isotherms at 77K: as-synthesised and regenerated (R1) materials.

8. X-ray Pair Distribution Function Analysis

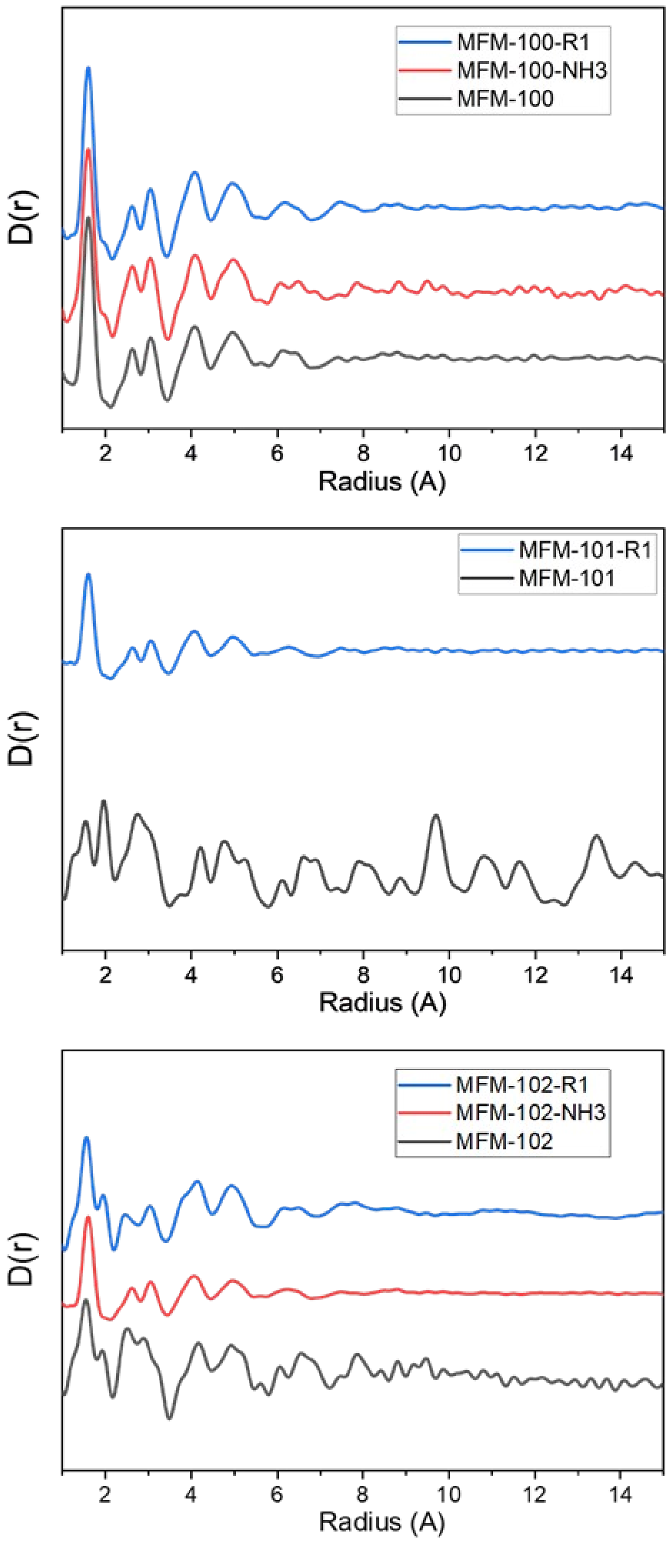

Figure S18. XPDF patterns of MFM-100, MFM-101 and MFM-102 (bare material, ammonia-loaded and regenerated materials) from the short-range to long-range region.

9. Electron Paramagnetic Resonance Spectroscopy

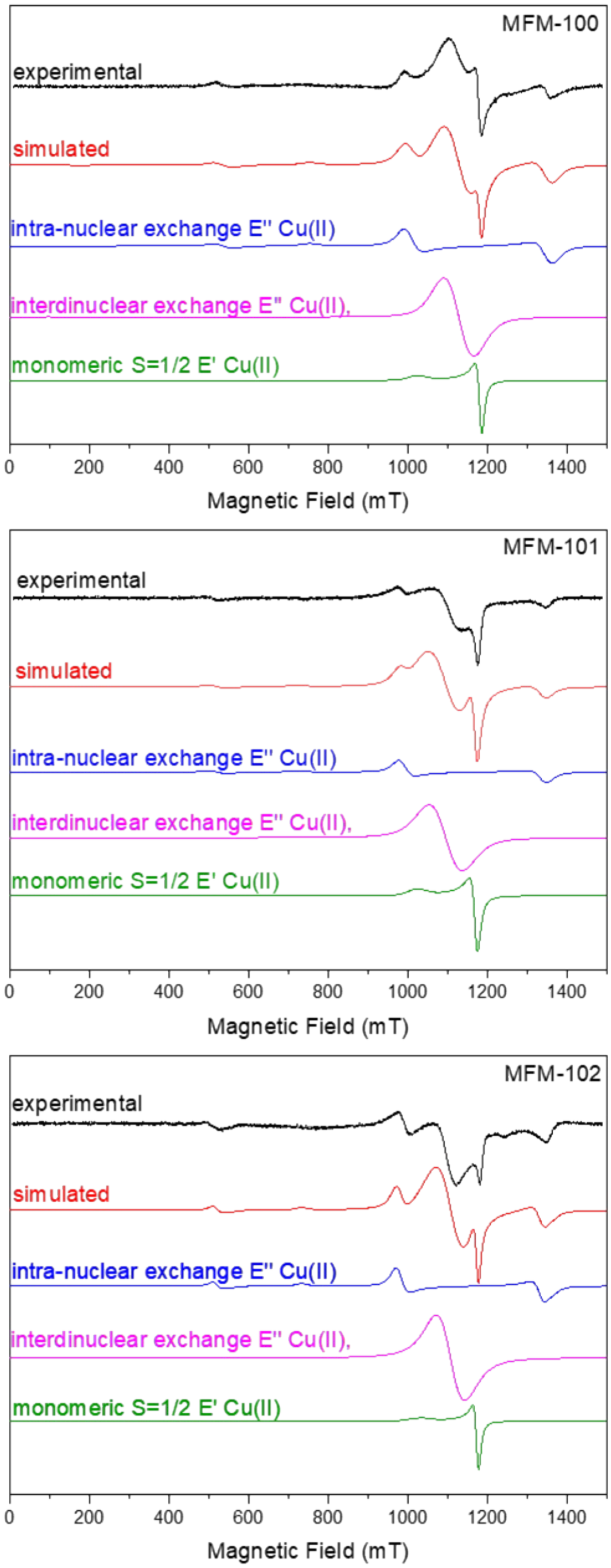

Figure S19. Q-band EPR spectra of as-synthesised MFM-100, MFM-101 and MFM-102 at 150 K, showing the superposition of the spectra of different Cu(II) species

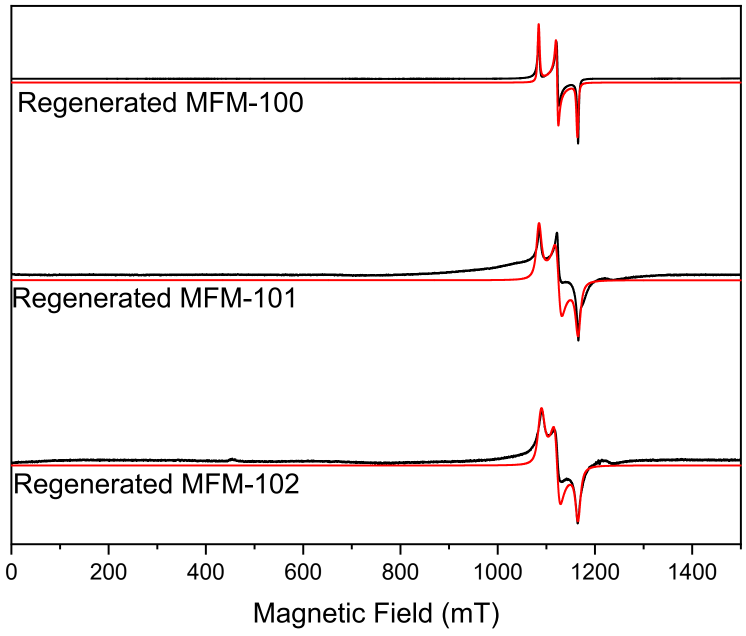

Figure S20. Q-band EPR spectra of regenerated MFM-100, MFM-101 and MFM-102 at 150 K.

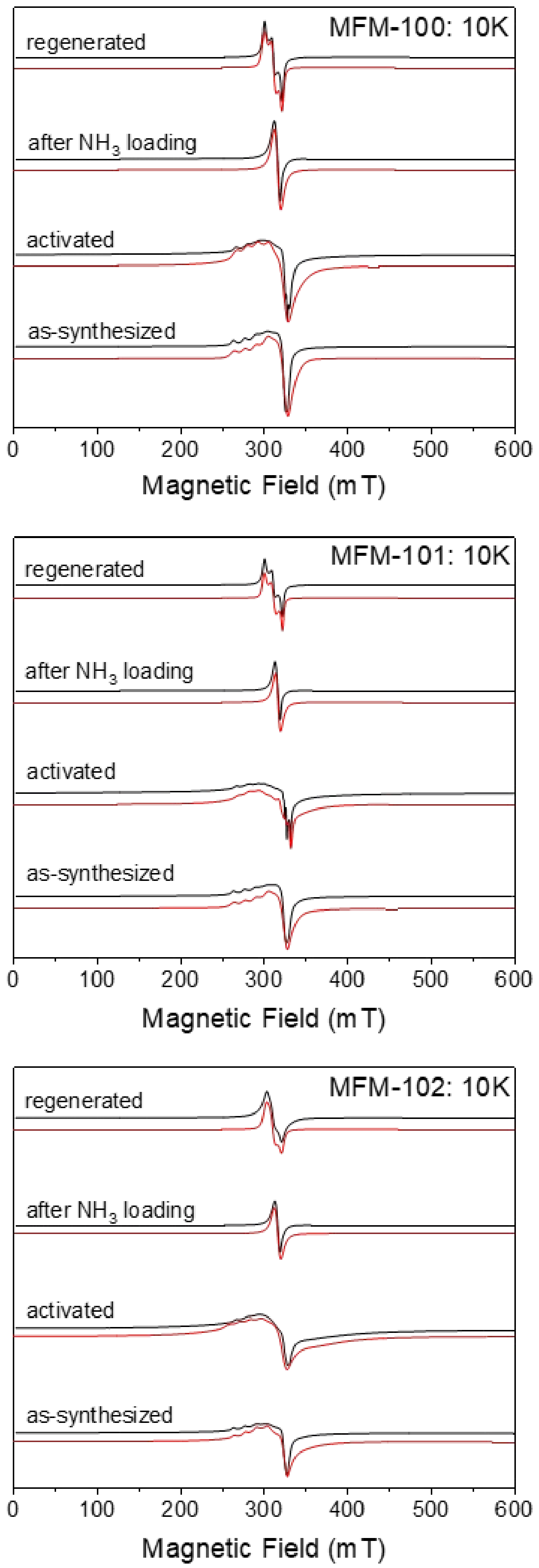

Figure S21. X-band EPR spectra of MFM-100, MFM-101 and MFM-102 at 10 K (experimental: black; simulated: red).

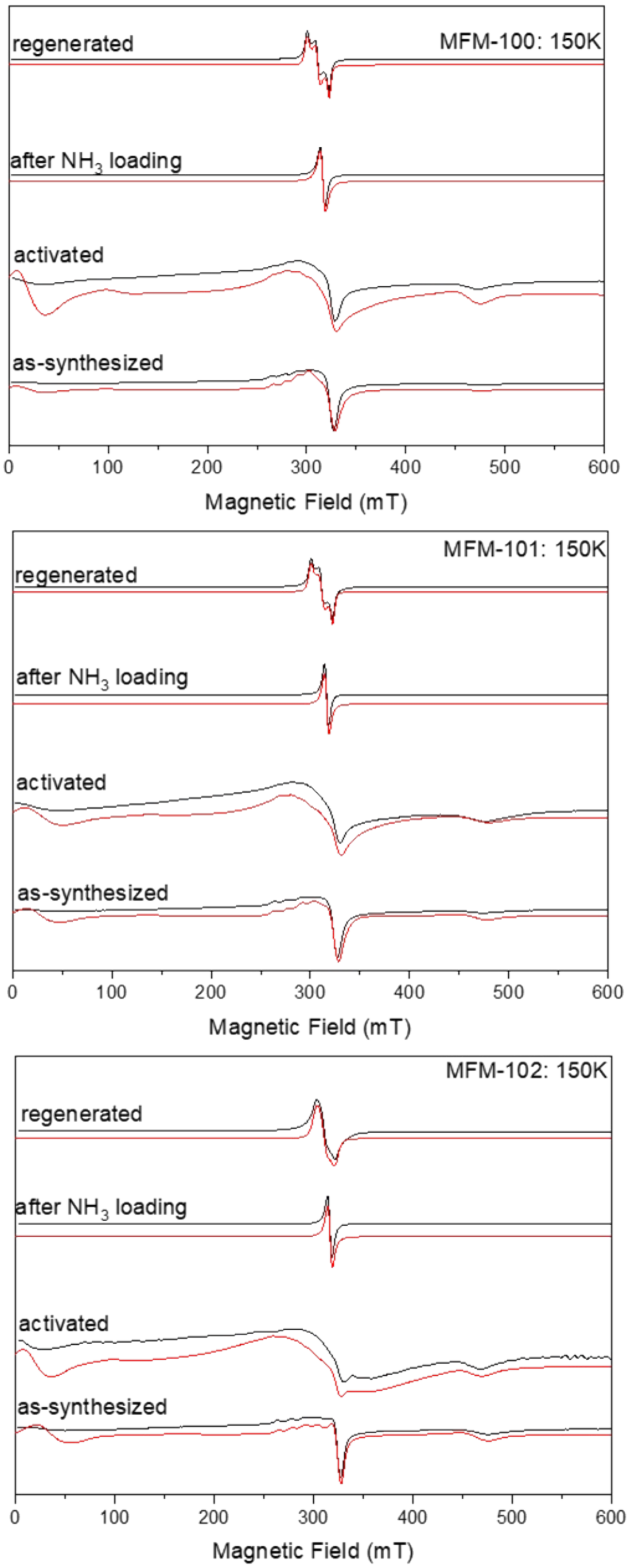

Figure S22. X-band EPR spectra of MFM-100, MFM-101 and MFM-102 at 150 K (experimental: black; simulated: red).

Note: No spin echo signal was detected for the NH_3-loaded and regenerated samples, due we believe to rapid relaxation of spins.

Table S2a. Spin-Hamiltonian parameter set extracted from Q-band CW EPR spectra of as-synthesized MFM-100, MFM-101 and MFM-102.

	Intra-nuclear exchange E'' Cu(II)	Interdinuclear exchange E'' Cu(II)	Monomeric E' Cu(II)
MFM-100	g = [2.05, 2.3]<br>D = -0.33 cm^{-1}<br>E = 0 cm^{-1}	g = [2.05, 2.3]<br>J_{intre} = 1 cm^{-1}	g = [2.063, 2.385]<br>gStrain = [0 0.1]
MFM-101	g = [2.07, 2.38]<br>D = -0.338 cm^{-1}<br>E = 0 cm^{-1}	g = [2.07, 2.38]<br>J_{intre} = 2 cm^{-1}	g = [2.082, 2.385]<br>gStrain = [0 0.1]
MFM-102	g = [2.09, 2.33]<br>D = -0.345 cm^{-1}<br>E = 0 cm^{-1}	g = [2.085, 2.385]<br>J_{intre} = 2 cm^{-1}	g = [2.063, 2.385]<br>gStrain = [0 0.1]

Table S2b. Spin-Hamiltonian parameter set extracted from Q-band CW EPR spectra of regenerated MFM-100, MFM-101 and MFM-102.

MFM-100	*g*= [2.178, 2.108, 2.26]<br>*gStrain* = [0.02, 0.0, 0.025];
MFM-101	*g*= [2.16, 2.085, 2.24]<br>*gStrain* = [0.01, 0.0, 0.0];
MFM-102	*g*= [2.165, 2.085, 2.243]<br>*gStrain* = [0.01, 0.0, 0.015];

*The names attributed to the three paramagnetic centres follow the usual convention: "E" indicates that the paramagnetic centre involves unpaired electrons; the number of apostrophes " ' " indicates the number of unpaired electrons actually involved in the centre.

Table S3. Spin-Hamiltonian parameter set extracted from X-band CW EPR spectra of as-synthesized, activated, NH_3-loaded and regenerated MFM-100, MFM-101 and MFM-102.

	MFM-100	MFM-101	MFM-102
as-synthesized	g= [2.07, 2.3]<br>D= -0.33 cm^{-1}<br>E=0 cm^{-1}	g= [2.07, 2.38]<br>D= -0.338 cm^{-1}<br>E=0 cm^{-1}	g= [2.09, 2.32]<br>D= -0.345 cm^{-1}<br>E=0 cm^{-1}
	g= [2.09, 2.32]<br>J'=1 cm^{-1}	g= [2.07, 2.38]<br>J'=1 cm^{-1}	g= [2.09, 2.32]<br>J'=2 cm^{-1}
	g=[2.09, 2.385]<br>A_z= 400 MHz<br>*gStrain*=[0.1 0]	g=[2.094, 2.385]<br>A_z= 440 MHz<br>*gStrain*=[0 0]	g=[2.095, 2.385]<br>A_z= 440 MHz<br>*gStrain*=[0.01 0]
activated	g= [2.07, 2.3]<br>D= -0.33 cm^{-1}<br>E=0 cm^{-1}	g= [2.07, 2.38]<br>D= -0.338 cm^{-1}<br>E=0 cm^{-1}	g= [2.09, 2.32]<br>D= -0.33 cm^{-1}<br>E=0 cm^{-1}
	g= [2.07, 2.3]<br>J'=1 cm^{-1}	g= [2.07, 2.38]<br>J'=1 cm^{-1}	g= [2.09, 2.32]<br>J'=2 cm^{-1}
	g=[2.09, 2.09, 2.37]<br>A_z= 430 MHz<br>*gStrain*=[0.17, 0.03, 0.06]	g=[2.08, 2.08, 2.35]<br>A_z= 420 MHz<br>*gStrain*=[0.17, 0.03, 0.06]	g=[2.1, 2.1, 2.44]<br>A_z= 430 MHz<br>*gStrain*=[0.17, 0.03, 0.08]
NH_3 loaded	g=2.144	g=2.141	g=2.144
regenerated	g= [2.178, 2.108, 2.26]<br>*gStrain* = [0.02, 0.0, 0.025];	g= [2.179, 2.105, 2.26]<br>*gStrain* = [0.03, 0.0, 0.025];	g= [2.186, 2.11, 2.243]<br>*gStrain* = [0.02, 0.0, 0.025];

*J'= the exchange coupling constant. The simulation of the interdinuclear exchange line is following with the previous research, of which only one interdinuclear exchange path with the exchange coupling constant J' was considered.[41]

10. X-ray Photoelectron Spectroscopy

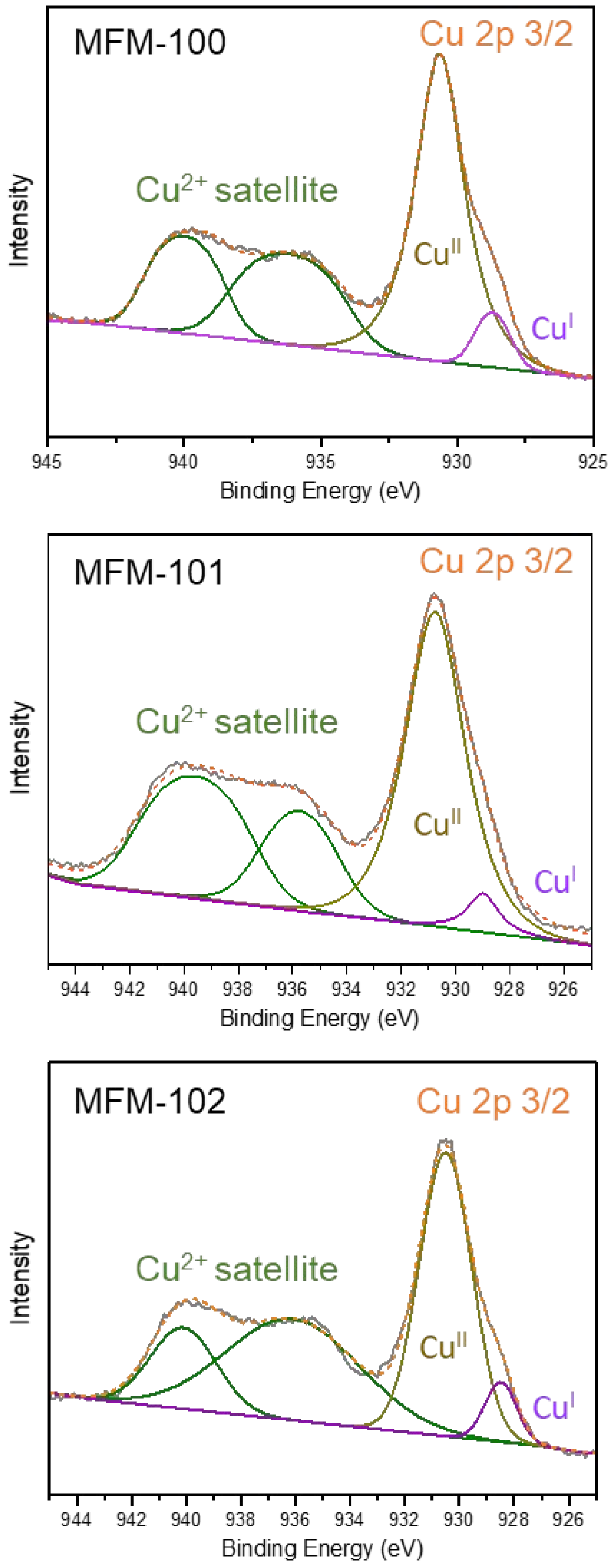

Figure S23. X-ray photoelectron spectroscopy (XPS) spectra of MFM-100, MFM-101 and MFM-102. A charge neutraliser was used to minimise charging and spectra are aligned on the binding energy scale relative to the hydrocarbon C-C/C-H peak at 284.8 eV. Spectra were fitted using the CASA XPS software using Voigt-like peak shapes. Spin-orbit splitting ratios and splitting energies are constrained to obtain physically meaningful fits.

11. Comparison of Ammonia Adsorption Capacity in MOFs

Table S4. Summary of the isothermal adsorption capacities for NH_3 and details of selected MOF materials.

Name	Uptake (mmol g^{-1})	Measuring Condition	Stability towards Dry Ammonia	Reference
Mg_2(dobpdc)	23.9	298K 1bar	Reversible for 3 Cycles	10
Ni_acryl_TMA	23.5	298K 1bar	Reversible for 5 Cycles	43
**MFM-101**	**21.9**<br>**14.9**	**273K 1bar**<br>**298K 1bar**	**Loss of Crystallinity**	**This work**
**MFM-127**	**21.5**<br>**14.8**	**273K 1bar**<br>**298K 1bar**	**Loss of Crystallinity**	**This work**
Ni_2(dobpdc)	20.8	298K 1bar	Reversible for 3 Cycles	10
Cu@Th-BPYDC	20.6	298K 1bar	Reversible for 3 Cycles	39
**MFM-102**	**20.4**<br>**13.7**	**273K 1bar**<br>**298K 1bar**	**Loss of Crystallinity**	**This work**
Cu_2Cl_2BBTA	19.8	298K 1bar	Loss of Crystallinity	11
**MFM-100**	**19.8**<br>**15.7**	**273K 1bar**<br>**298K 1bar**	**Loss of Crystallinity**	**This work**
Co_2Cl_2BBTA	17.9	298K 1bar	Reversible for 3 Cycles	11
Fe-MIL-101-SO_3H	17.8	298K 1bar	Not Discussed	12
$Zn_2(L_1)_2$(bpe)	17.8	293K 1bar	Not Discussed	13
$Co(NA)_2$	17.5	298K 1bar	Loss of Crystallinity	14
Ni_acryl_TGA	17.4	298K 1bar	Reversible for 5 Cycles	43
MFM-300(V^{IV})	17.3	273K 1bar	Reversible for 18 cycles	15
**MFM-170**	**17.2**<br>**13.6**	**273K 1bar**<br>**298K 1bar**	**Loss of Crystallinity**	**This work**
UiO-66-Cu^{II}	16.9	273K 1bar	Reversible for 15 cycles	35
**MFM-190(F)**	**16.8**<br>**12.7**	**273K 1bar**<br>**298K 1bar**	**Loss of Crystallinity**	**This work**
DUT-6-$(OH)_2$	16.4	298K 1bar	Loss of Crystallinity	16

Mg-MOF-74	16.2	298K 1bar	Stable up to 350 °C	17
MFM-300(V^{III})	16.1	273K 1bar	Reversible for 24 cycles	15
**MFM-126**	**15.9**<br>**11.8**	**273K 1bar**<br>**298K 1bar**	**Loss of Crystallinity**	**This work**
MFM-300(Al)	15.7	273K 1bar	Reversible for 50 cycles	18
MFM-300(Fe)	15.6	273K 1bar	Reversible for 20 cycles	15
Mn_2Cl_2BTDD	15.5	298K 1bar	Reversible for 3 Cycles	19
Zn_2(dobpdc)	15.2	298K 1bar	Loss of Crystallinity	10
Fe-soc-MOF	14.7	298K 1bar	Decrease of Uptake	20
Ni_2Cl_2BBTA	14.7	298K 1bar	Reversible for 3 Cycles	11
UiO-67-PSF-Mn	14.5	298K 1.3bar	Retained Crystallinity	38
$Zn_2(L_1)_2$(bipy)	14.3	293K 1bar	Not Discussed	13
**Cu-MOP-1a**	**14.2**<br>**11.6**	**273K 1bar**<br>**298K 1bar**	**Loss of Crystallinity**	**This work**
MFM-300(Cr)	14.0	273K 1bar	Reversible for 35 cycles	15
$Cu(NA)_2$	13.4	298K 1bar	Loss of Crystallinity	14
Co_2(dobpdc)	13.3	298K 1bar	Reversible for 3 Cycles	13
Mn_2(dobpdc)	13.3	298K 1bar	Reversible for 3 Cycles	13
Ni_acrylate	13.1	298K 1bar	Reversible for 5 Cycles	43
MFM-300(Sc)	13.1	298K 1bar	Reversible for 5 Cycles	34
UiO-66-Cu^I	12.6	273K 1bar	Reversible for 15 cycles	35
MOF-5	12.2	298K 1bar	Loss of Crystallinity	21
MOF-177	12.2	298K 1bar	Loss of Crystallinity	21
HKUST-1	12.1	301K, 1.2 bar	Loss of Crystallinity	22
$Ni_2(adc)_2$(dabco)	12.1	295K 1bar	Reversible for 3 Cycles	23

Ni_2Cl_2BTDD	12.0	298K 1bar	Reversible for 3 Cycles	19
Co_2Cl_2BTDD	12.0	298K 1bar	Loss of Crystallinity	19
DUT-6	12.0	298K 1bar	Loss of Crystallinity	16
UiO-66-defect	11.8	273K 1bar	Reversible for 15 cycles	35
$Co_2(adc)_2(dabco)$	11.2	295K 1bar	Reversible for 3 Cycles	23
Ga-PMOF	10.5	298K 1bar	Loss of Crystallinity	24
UiO-67-ox-Cu	10.5	298K 1bar	Retained Crystallinity	37
PFC-27/CF_3SO_3	10.5	298K 1.3 bar	Retained Crystallinity	36
$Zn(NA)_2$	10.2	298K 1bar	Structural Change	14
MIL-101	10.0	298K 1bar	Reversible for 5 Cycles	25
MFM-303	9.9	273K 1bar	Reversible for 30 Cycles	40
In-PMOF	9.4	298K 1bar	Decrease of Uptake	24
UiO-bpydc	8.4	298K 1.1bar	Stable for 1 Cycle	26
NU-1401	8.4	298K 1bar	Stable for 1 Cycle	27
UiO-67	8.4	298K 1.1bar	Stable for 1 Cycle	26
$Zn_2(adc)_2(dabco)$	8.3	295K 1bar	Reversible for 3 Cycles	23
NU-300	8.3	298K 1bar	Loss of Crystallinity	28
UiO-66-C	8.3	298K 1bar	Stable for 1 Cycle	29
MIL-100	8.0	298K 1bar	Reversible for 5 Cycles	25
NU-1000-Cl-120	7.8	298K 1bar	Reversible for 3 Cycles	30
NU-1000-F-120	7.8	298K 1bar	Reversible for 3 Cycles	34
Al-PMOF	7.7	298K 1bar	Reversible for 2 Cycles	24
NU-1000-F-60	7.6	298K 1bar	Reversible for 3 Cycles	31
NU-1000-Cl-300	7.1	298K 1bar	Reversible for 3 Cycles	30

UiO-67-vac	7.1	298K 1.3bar	Retained Crystallinity	37
NDC-NU-1000-F-120	6.9	298K 1bar	Reversible for 3 Cycles	31
UiO-66-B	6.8	298K 1bar	Stable for 1 Cycle	29
NDC-NU-1000-F-60	6.8	298K 1bar	Reversible for 3 Cycles	31
ECUT-36	6.6	273K 1bar	Reversible for 2 Cycles	32
$Cu_2(adc)_2(dabco)$	6.5	295K 1bar	Loss of Crystallinity	23
NDC-NU-1000-FF-60	6.2	298K 1bar	Reversible for 3 Cycles	31
$Cd(NA)_2$	6.0	298K 1bar	Structural Change	14
Al-BTB	6.0	298K 1bar	Stable up to 350 °C	17
NU-1000-FF-300	5.9	298K 1bar	Reversible for 3 Cycles	31
NU-1000-FF-60	5.8	298K 1bar	Reversible for 3 Cycles	31
NDC-NU-1000-FF-120	5.8	298K 1bar	Reversible for 3 Cycles	38
UiO-66-A	5.7	298K 1bar	Stable for 1 Cycle	29
SION105-Eu	5.7	303K 1bar	Reversible for 5 Cycles	33
NU-1000-FF-120	5.5	298K 1bar	Reversible for 3 Cycles	31
NU-1000-F-300	5.4	298K 1bar	Reversible for 3 Cycles	31
NH_2-MIL-53	5.4	298K 1bar	Reversible for 5 Cycles	25
MIL-53	4.4	298K 1bar	Reversible for 5 Cycles	25

Table S5. Summary of the dynamic adsorption capacities for dry NH_3 and details of selected MOF materials.

Name	Uptake (mmol/g)	Measuring Condition	Reference
Co_2Cl_2BBTA	8.56	298 K 1.0 mbar	11
Mg_2(dobpdc)	8.25	298 K 0.57 mbar	10
Cu_2Cl_2BBTA	7.52	298 K 1.0 mbar	11
IRMOF-3	6.16	298 K 0.99 mbar	42
Ni_2(dobpdc)	5.16	298 K 0.58 mbar	10
MOF-199	5.11	298 K 0.99 mbar	42
Zn_2(dobpdc)	4.98	298 K 0.42 mbar	10
Co_2Cl_2BTDD	4.78	298 K 1.0 mbar	11
Mn_2(dobpdc)	4.77	298 K 0.36 mbar	10
Co_2(dobpdc)	4.72	298 K 0.54 mbar	10
**MFM-102**	**4.50**	**298 K 1.0 mbar**	**This work**
UiO-66-Cu^{II}	4.15	298 K 0.63 mbar	35
Ni_acryl_TMA	4.11	298 K 1.0 mbar	43
**MFM-101**	**4.09**	**298 K 1.0 mbar**	**This work**
Fe-MIL-101-SO_3H	3.52	298 K 0.51 mbar	12
Ni_2Cl_2BTDD	3.36	298 K 1.0 mbar	43
**MFM-100**	**3.32**	**298 K 1.0 mbar**	**This work**
Ni_acryl_TGA	3.09	298 K 1.0 mbar	43
UiO-66-Cu^{I}	3.07	298 K 0.63 mbar	35
MFM-303	2.9	298K 0.83 mbar	40
UiO-66-NH_3Cl	2.64	298 K 0.66 mbar	12
MOF-177	2.47	298 K 0.99 mbar	42

UiO-66-defect	2.07	298K 0.63mbar	35
Ni_acrylate	1.97	298 K 1.0 mbar	43
MFM-300(V^{III})	1.9	298 K 1.0 mbar	15
IRMOF-62	1.35	298 K 0.99 mbar	42
MFM-300(Cr)	1.1	298 K 1.0 mbar	15
MFM-300(V^{IV})	1.0	298 K 1.0 mbar	15
UiO-66-NH_2	0.93	298 K 0.49 mbar	12
MFM-300(Fe)	0.6	298 K 1.0 mbar	15
MOF-5	0.35	298 K 0.99 mbar	42

Table S6. Summary of the NH_3 storage density and packing density of selected MOF materials.

Name	Condition	Storage density (g/cm^3)	Packing density (g/cm^3)	Reference
Ni_acryl_TMA	298 K, 1 bar	0.39	0.70	43
**MFM-127**	**273 K, 1 bar**	**0.37**	**0.70**	**This work**
**MFM-126**	**273 K, 1 bar**	**0.36**	**0.75**	**This work**
**MFM-102**	**273 K, 1 bar**	**0.35**	**0.52**	**This work**
MFM-300(V^{IV})	273 K, 1 bar	0.34	0.61	15
UiO-66-Cu^{II}	273 K, 1 bar	0.34	0.74	35
**MFM-101**	**273 K, 1 bar**	**0.33**	**0.58**	**This work**
**MFM-100**	**273 K, 1 bar**	**0.33**	**0.58**	**This work**
MFM-300(Fe)	273 K, 1 bar	0.30	0.60	15
MFM-300(V^{III})	273 K, 1 bar	0.29	0.54	15
MFM-300(Al)	273 K, 1 bar	0.28	0.72	15
MFM-300(Cr)	273 K, 1 bar	0.27	0.51	15
Ni_acryl_TGA	298 K, 1 bar	0.26	0.52	43
UiO-66-Cu^{I}	273 K, 1 bar	0.25	0.55	35
**Cu-MOP-1a**	**273 K, 1 bar**	**0.25**	**0.79**	**This work**
UiO-66-defect	273 K, 1 bar	0.24	0.52	35
Ni_2(dobpdc)	298 K, 1 bar	0.23	0.31	10
$Zn_2(L_1)_2$(bpe)	293 K, 1 bar	0.23	Not Discussed	13
$Zn_2(L_1)_2$(bipy)	293 K, 1 bar	0.23	Not Discussed	13
Mg_2(dobpdc)	298 K, 1 bar	0.22	0.25	10
MFM-300(Sc)	298 K, 1 bar	0.22	Not Discussed	34
**MFM-190(F)**	**273 K, 1 bar**	**0.22**	**0.35**	**This work**

Ga-PMOF	298 K, 1 bar	0.21	0.37	24
**MFM-170**	**273 K, 1 bar**	**0.21**	**0.34**	**This work**
NU-1401	298 K, 1 bar	0.19	0.62	27
Mn_2Cl_2BTDD	298 K, 1 bar	0.18	0.19	19
Zn_2(dobpdc)	298 K, 1 bar	0.18	0.24	10
HKUST-1	301 K, 1.2 bar	0.18	Not Discussed	22
Ni_acrylate	298 K, 1 bar	0.16	0.24	43
Ni_2Cl_2BTDD	298 K, 1 bar	0.16	0.18	43
Co_2(dobpdc)	298 K, 1 bar	0.15	0.21	10
Mn_2(dobpdc)	298 K, 1 bar	0.15	0.19	10
NU-300	298 K, 1 bar	0.13	0.24	28
SION105-Eu	298 K, 1 bar	0.13	Not Discussed	33
MOF-5	298 K, 1 bar	0.12	0.14	21
MIL-100	298 K, 1 bar	0.1	Not Discussed	25
ECUT-36	298 K, 1 bar	0.1	0.76	32
MOF-177	298 K, 1 bar	0.06	0.07	21

References

[1] Lin, X.; Jia, J.; Zhao, X.; Thomas, K. M.; Blake, A. J.; Walker, G. S.; Champness, N.R.; Hubberstey, P.; Schröder, M. High H_2 adsorption by coordination-framework materials. *Angew. Chem.* **2006**, 118, 7518–7524.

[2] Humby, J. D.; Benson, O.; Smith, G. L.; Argent, S.P.; Da Silva, I.; Cheng, Y.; Rudić, S.; Manuel, P.; Frogley, M. D.; Cinque, G.; Saunders, L. K.; Vitórica-Yrezábal, I. J.; Whitehead, G. F. S.; Easun, T. L.; Lewis, W.; Blake, A. J.; Ramirez-Cuesta, A. J.; Yang, S.; Schröder, M. Host–guest selectivity in a series of isoreticular metal-organic frameworks: observation of acetylene-to-alkyne and carbon dioxide-to-amide interactions. *Chem. Sci.* **2019**, 10, 1098–1106.

[3] Argent, S. P.; Da Silva, I.; Greenaway, A.; Savage, M.; Humby, J.; Davies, A. J.; Nowell, H.; Lewis, W.; Manuel, P; Tang, C. C.; Blake, A. J.; George. M. W.; Markevich, A. V.; Besley, E.; Yang, S.; Champness, N.R.; Schröder, M. Porous metal-organic polyhedra: morphology, porosity, and guest binding. *Inorg. Chem.* **2020**, 59, 15646–15658.

[4] Smith, G. L.; Eyley, J. E.; Han, X.; Zhang, X.; Li, J.; Jacques, N. M.; Godfrey, H. G.; Argent, S. P.; McPherson, L. J.; Teat, S. J.; Cheng, Y.; Frogley, M. D.; Cinque, G.; Day, S. J.; Tang, C. C.; Easun, T. L.; Rudić, S.; Ramirez-Cuesta, A. J.; Yang, S.; Schröder, M. Reversible coordinative binding and separation of sulfur dioxide in a robust metal-organic framework with open copper sites. *Nat. Mater.* **2019**, 18, 1358–1365.

[5] Li, W.; Li, J.; Duong, T. D.; Sapchenoko, S. A.; Han, X.; Humby, J. D.; Whitehead, F. S. G.; Victórica-Yrezábal, I. J.; da Silva, I.; Manuel, P.; Frogley, M.D.; Cinque, G.; Schröder, M.; Yang, S. Adsorption of sulphur dioxide in Cu(II)-carboxylate framework materials: the role of ligand functionalisation and open metal sites. *J. Am. Chem. Soc.* **2022**, 144, 13196–13204.

[6] Glomb, S.; Woschko, D.; Makhloufi, G.; Janiak, C. Metal-organic frameworks with internal urea-functionalized dicarboxylate linkers for SO_2 and NH_3 desorption. *ACS Appl. Mater. Interfaces.* **2017**, 9, 37419–37434.

[7] Soper, A. K.; Howells, S.; Hannon, A. C. ATLAS: Analysis of time-of-flight diffraction data from liquid and amorphous samples; RAL Report RAL-89-046; Rutherford Appleton Laboratory: Chilton, Didcot, Oxon, U.K., 1999.

[8] Farrow, C. L.; Juhas, P.; Liu, J. W.; Bryndin, D.; Božin, E. S.; Bloch, J.; Proffen, T.; Billinge, S. J. L. *J. Phys.: Condens. Matter* **2007**, 19, 335219.

[9] Stoll, S. & Schweiger, A. EasySpin, a comprehensive software package for spectral simulation and analysis in EPR. *J. Magn. Reson.* **2006**, 178, 42–55.

[10] Kim, D. W.; Kang, D. W.; Kang, M.; Lee, J.; Choe, J. H.; Chae, Y. S.; Choi, D. S.; Yun, H.; Hong, C. S. High ammonia uptake of a metal-organic framework adsorbent in a wide pressure range. *Angew. Chem. Int. Ed.* **2020**, 59, 22531–22536.

[11]Rieth, A. J.; Dincă, M. Controlled gas uptake in metal-organic frameworks with record ammonia sorption. *J. Am. Chem. Soc*. **2018**, 140, 3461–3466.

[12] Van Humbeck, J. F.; McDonald, T. M.; Jing, X.; Wiers, B. M.; Zhu, G.; Long, J. R. Ammonia capture in porous organic polymers densely functionalized with Brønsted acid groups. *J. Am. Chem. Soc.* **2014,** 136, 2432–2440.

[13] Glomb, S.; Woschko, D.; Makhloufi, G.; Janiak, C. Metal-organic frameworks with internal urea-functionalized dicarboxylate linkers for SO_2 and NH_3 desorption. *ACS Appl. Mater. Interfaces.* **2017**, 9, 37419–37434.

[14] Chen, Y.; Shan, B.; Yang, C.; Yang, J.; Li, J.; Mu, B. Environmentally friendly synthesis of flexible MOFs $M(NA)_2$ (M= Zn, Co, Cu, Cd) with large and regenerable ammonia capacity. *J. Mater. Chem. A.* **2018**, 6, 9922–9929.

[15] Han, X.; Lu, W.; Chen, Y.; da Silva, I.; Li, J.; Lin, L.; Li, W.; Sheveleva, A. M.; Godfrey, H. G. W.; Lu, Z.; Tuna, F.; McInnes, E. J. L.; Cheng, Y.; Daemen, L. L.; McPherson, L. J. M.; Teat, S. J.; Frogley, M. D.; Rudic, S.; Manuel, P.; Ramirez-Cuesta, A. J.; Yang, S.; Schroder, M. High ammonia adsorption in MFM-300 materials: dynamics and charge transfer in host-guest binding. *J. Am. Chem. Soc.* **2021**, 143, 3153–3161.

[16] Spanopoulos, I.; Xydias, P.; Malliakas, C. D.; Trikalitis, P. N. A straight-forward route for the development of metal-organic frameworks functionalized with aromatic -OH groups: synthesis, characterization, and gas (N_2, Ar, H_2, CO_2, CH_4, NH_3) sorption properties. *Inorg. Chem*. **2013**, 52, 855–862.
[17] Kajiwara, T.; Higuchi, M.; Watanabe, D.; Higashimura, H.; Yamada, T.; Kitagawa, H. A systematic study on the stability of porous coordination polymers against ammonia. *Chem. Eur. J.* **2014**, 20, 15611–15617.
[18] Godfrey, H. G. W.; da Silva, I.; Briggs, L.; Carter, J. H.; Morris, C. G.; Savage, M.; Easun, T. L.; Manuel, P.; Murray. C. A.; Tang, C. C.; Frogley. M. D.; Cinque, G.; Yang, S.; Schröder, M. Ammonia storage by reversible host-guest site exchange in a robust metal-organic framework. *Angew. Chem. Int. Ed.* **2018**, 57, 14778–14781.
[19] Rieth, A. J.; Tulchinsky, Y.; Dincă, M. High and reversible ammonia uptake in mesoporous azolate metal–organic frameworks with open Mn, Co, and Ni sites. *J. Am. Chem. Soc*. **2016**, 138, 9401–9404.
[20] Chen, Z.; Wang, X.; Cao, R.; Idrees, K. B.; Liu, X.; Wasson, M.; Farha, O. K. Water-based synthesis of a stable iron-based metal-organic framework for capturing toxic gases. *ACS Materials Lett.* **2020**, 2, 1129–1134.
[21] Saha, D.; Deng, S.; Ammonia adsorption and its effects on framework stability of MOF-5 and MOF-177. *J. Colloid Interface Sci.* **2010**, 348, 615–620.
[22] Petit, C.; Huang, L.; Jagiello, J.; Kenvin, J.; Gubbins, K. E.; Bandosz, T. J. Toward understanding reactive adsorption of ammonia on Cu-MOF/graphite oxide nanocomposites. *Langmuir.* **2011**, 27, 13043–13051.
[23] Cao, Z.; Landström, K. N.; Akhtar, F. Rapid ammonia carriers for CR systems using MOFs $[M_2(adc)_2(dabco)]$ (M=Co, Ni, Cu, Zn). *Catalysts.* **2020**, 10, 1444–1454.
[24] Moribe, S.; Chen, Z.; Alayoglu, S.; Syed, Z. H.; Islamoglu, T.; Farha, O. K. Ammonia capture within isoreticular metal─organic Frameworks with rod secondary building units. *ACS Mater. Lett.* **2019**, 1, 476–480.
[25] Chen, Y.; Zhang, F.; Wang, Y.; Yang, C.; Yang, J.; Li, J. Recyclable ammonia uptake of a MIL series of metal-organic frameworks with high structural stability. *Microporous Mesoporous Mater.* **2018**, 258, 170–177.
[26] Yoskamtorn, T.; Zhao, P.; Wu, X. P.; Purchase, K.; Orlandi, F.; Manuel, P.; Taylor, J.; Li, Y.; Day, S.; Ye, L.; Tang, C. C.; Responses of defect-rich Zr-based metal-organic frameworks toward NH_3 adsorption. *J. Am. Chem. Soc.* **2021**, 143, 3205–3218.
[27] Zhang, Y.; Zhang, X.; Chen, Z.; Otake, K.; Peterson, G. W.; Chen, Y.; Wang, X.; Redfern, L. R.; Goswami, S.; Li, P.; Islamoglu, T.; Wang, B.; Farha, O. K. A Flexible interpenetrated zirconium-based metal-organic framework with high affinity toward ammonia. *ChemSusChem*. **2020**, 13, 1710–1714.
[28] Chen, Y.; Zhang, X.; Ma, K.; Chen, Z.; Wang, X.; Knapp, J.; Alayoglu, S.; Wang, F.; Xia, Q.; Li, Z.; Islamoglu, T.; Farha, O. K. Zirconium-based metal-organic framework with 9-connected nodes for ammonia capture. *ACS Appl. Nano Mater*. **2019**, 2, 6098–6102.
[29] Morris, W.; Doonan, C. J.; Yaghi, O. M. Postsynthetic modification of a metal–organic framework for stabilization of a hemiaminal and ammonia uptake. *Inorg. Chem.* **2011**, 50, 6853–6855.
[30] Liu, J.; Chen, Z.; Wang, R.; Alayoglu, S.; Islamoglu, T.; Lee, S.; Sheridan, T. R.; Chen, H.; Snurr, R. Q.; Farha, O. K.; Hupp, J. T. Zirconium metal-organic frameworks integrating chloride ions for ammonia capture and/or chemical separation. *ACS Appl. Mater. Interfaces.* **2021**, 13, 22485–22494.
[31] Liu, J.; Lu, Z.; Chen, Z.; Rimoldi, M.; Howarth, A. J.; Chen, H.; Alayoglu, S.; Snurr, R. Q.; Farha, O. K.; Hupp, J. T. Ammonia capture within zirconium metal-organic frameworks: reversible and irreversible uptake. *ACS Appl. Mater. Interfaces.* **2021**, 13, 20081–20093.

[32] Sun, L. J.; Fan, Y. L.; Yin, M. J.; Zhang, H. P.; Feng, H.; Guo, L. J.; Luo, F. Thorium metal-organic framework showing proton transformation from $[NH_2(CH_3)_2]^+$ to the carboxyl group to enhance porosity for selective adsorption of D_2 over H_2 and ammonia capture. *Cryst. Growth Des.* **2020**, 20, 3605–3610.

[33] Nguyen, T. N.; Harreschou, I. M.; Lee, J. H.; Stylianou, K. C.; Stephan, D. W. A recyclable metal-organic framework for ammonia vapour adsorption. *Chem. Commun*. **2020**, 56, 9600.

[34] Lyu, P.; Wright, A. M.; López-Olvera, A.; Mileo, P. G.; Zárate, J. A.; Martínez-Ahumada, E.; Martis, V.; Williams, D. R.; Dincă, M.; Ibarra, I. A.; Maurin, G.; Ammonia capture via an unconventional reversible guest-induced metal-linker bond dynamics in a highly stable metal-organic framework. *Chem. Mater.* **2021**. 33, 6186–6192.

[35] Ma, Y.; Lu, W.; Han, X.; Chen, Y.; da Silva, I.; Lee, D.; Sheveleva, A. M.; Wang, Z.; Li, J.; Li, W.; Fan, M.; Xu, S.; Tuna, F.; McInnes, J. L. E.; Cheng, Y.; Rudic, S.; Manuel, P.; Frogley, M. D.; Ramirez-Cuesta, A. J.; Schroder, M.; Yang, S. Direct observation of ammonia storage in UiO-66 incorporating Cu(II) binding sites. *J. Am. Chem. Soc.* **2022**, 144, 8624−8632.

[36] Matikolaei, M.; K.; Binaeian, E. Boosting ammonia uptake within metal-organic frameworks by anion modulating strategy. *ACS Appl. Mater. Interfaces* **2021**, 13, 27159–27168.

[37] Binaeian, E.; Li, Y.; Tayebi, H.; Yuan, D. Enhancing toxic gas uptake performance of Zr-based MOF through uncoordinated carboxylate and copper insertion; ammonia adsorption. *J. Hazard. Mater.* **2021**, 416, 125933.

[38] Binaeian, E.; Li, Y.; Yuan, D. Improving ammonia uptake performance of zirconium-based metal-organic frameworks through open metal site insertion strategy. *Chem. Eng. J.* **2021**, 421, 129655.

[39] Gao, Z.; Lai, Y.; Tao, Y.; Xiao, L.; Zhang, L.; Luo, F. Constructing well-defined and robust Th-MOF-supported single-site copper for production and storage of ammonia from electroreduction of nitrate. *ACS Cent. Sci.* **2021**, 7, 1066–1072.

[40] Marsh, C.; Han, X.; Li, J.; Lu, Z.; Argent, S. P.; Da Silva, I.; Cheng, Y.; Daemen, L. L.; Ramirez-Cuesta, A. J.; Thompson, S. P.; Blake, A. J.; Yang, S.; Schröder, M. Exceptional packing density of ammonia in a dual-functionalized metal–organic framework. *J. Am. Chem. Soc.* **2021**, 143, 6586–6592.

[41] Simenas, M.; Kobalz, M.; Mendt, M.; Eckold, P.; Krautscheid, H.; Banys, J.; Pöppl, A.; Synthesis, structure, and electron paramagnetic resonance study of a mixed valent metal–organic framework containing Cu_2 paddle-wheel units. *J. Phys. Chem. C* 2015, 119, 4898−4907.

[42] Britt, D.; Tranchemontagne, D.; Yaghi, O. M. Metal-organic frameworks with high capacity and selectivity for harmful gases. *Proc. Natl. Acad. Sci. U.S.A.* **2008**, 105, 11623–11627.

[43] Kim, D. W.; Kang, D. W.; Kang, M.; Choi, D. S.; Yun, H.; Kim, S. Y.; Lee, S. M.; Lee, S. M.; Hong, C. S. High gravimetric and volumetric ammonia capacities in robust metal–organic frameworks prepared via double postsynthetic modification. *J. Am. Chem. Soc.* **2022**, 144, 9672–968
